# Health benefits of leisure-time physical activity by socioeconomic status, lifestyle risk, and mental health: a multicohort study

**DOI:** 10.1016/S2468-2667(24)00300-1

**Published:** 2025-02-03

**Authors:** Solja T Nyberg, Philipp Frank, Jaana Pentti, Lars Alfredsson, Jenni Ervasti, Marcel Goldberg, Anders Knutsson, Aki Koskinen, Tea Lallukka, Maria Nordin, Ossi Rahkonen, Timo Strandberg, Sakari Suominen, Ari Väänänen, Jussi Vahtera, Marianna Virtanen, Hugo Westerlund, Marie Zins, Sari Stenholm, Séverine Sabia, Archana Singh-Manoux, Mark Hamer, Mika Kivimäki

**Affiliations:** a Clinicum, Department of Public Health, Faculty of Medicine, University of Helsinki, Helsinki, Finland; b Finnish Institute of Occupational Health, Helsinki, Finland; c UCL Brain Sciences, University College London, London, UK; d Division of Surgery & Interventional Science, Faculty of Medical Sciences, University College London, London, UK; e Department of Public Health, University of Turku and Turku University Hospital, Turku, Finland; f Centre for Population Health Research, University of Turku and Turku University Hospital, Turku, Finland; g Research Services, University of Turku and Turku University Hospital, Turku, Finland; h Institute of Environmental Medicine, Karolinska Institutet, Stockholm, Sweden; i Department of Clinical Neuroscience, Karolinska Institutet, Stockholm, Sweden; j Centre for Occupational and Environmental Medicine, Stockholm County Council, Sweden; k Epidemiology of Ageing and Neurodegenerative Diseases, U1153 Inserm, Université Paris Cité, Paris, France; l Population-based Cohorts Unit, Université Paris Cité, Paris, France; m UVSQ, UMS 011 Inserm, Paris Saclay University, Paris, France; n Department of Health Sciences, Mid Sweden University, Sundsvall, Sweden; o Stress Research Institute, University of Stockholm, Stockholm, Sweden; p Department of Psychology, Umeå University, Umeå, Sweden; q Helsinki University Hospital, Helsinki, Finland; r Department of Public Health, University of Turku and the Wellbeing Services County of Southwest Finland, Research Unit, Turku, Finland; s School of Health Science, University of Skövde, Skövde, Sweden; t School of Educational Sciences and Psychology, University of Eastern Finland, Joensuu, Finland

## Abstract

**Background:**

Regular physical activity is recommended for all aged 5 years and older, but the health benefits gained might differ across population subgroups. The aim of this study was to examine these benefits in terms of years lived free from major non-communicable diseases in subgroups with varying levels of risk factors.

**Methods:**

Our analysis was based on a multicohort study of initially healthy European adults from the IPD-Work Consortium and initially healthy participants from the UK Biobank study. Self-reported leisure-time physical activity levels at baseline (1986–2010) were categorised as low (no or very little), intermediate (between low and recommended levels), and WHO-recommended (≥2·5 h of moderate or ≥1·25 h of vigorous physical activity per week). We divided the study population into 36 overlapping subgroups based on socioeconomic factors, lifestyle, and mental health at baseline, and assessed disease-free years between ages 40 years and 75 years for both the overall population and subgroups, accounting for coronary heart disease, stroke, type 2 diabetes, cancer, asthma, and chronic obstructive pulmonary disease.

**Findings:**

14 IPD-Work studies were assessed and six studies were excluded due to missing outcome data and unavailable data for pooling, resulting in the inclusion of eight studies with 124 909 participants. After the exclusion of 7685 participants due to prevalent diseases and 9265 due to missing data, the sample consisted of 107 959 initially healthy European adults (63 567 [58·9%] females and 44 392 [41·1%] males) from the IPD-Work consortium. For the UK Biobank sample, 9 238 453 million individuals were invited, 8 736 094 (94·6%) were non-respondents, and 502 359 participated in the baseline examination. After the exclusion of 73 460 participants, 428 899 participants had data on at least one measure of physical activity. 236 258 (55·1%) were female and 192 641 (44·9%) were male. During 1·6 million person-years at risk, 21 231 IPD-Work participants developed a non-communicable disease, while 101 319 UK Biobank participants developed a non-communicable disease over 4·8 million person-years at risk. Compared with individuals with low physical activity, those meeting the recommended physical activity levels during leisure-time gained an additional 1·1 (95% CI 1·0–1·2) to 2·0 (1·7–2·3) disease-free years, depending on sex and study. In males from the IPD-Work and UK Biobank cohorts, greater gains in disease-free years were observed in current smokers (2·4 [95% CI 2·1–2·8]) versus never smokers (0·7 [0·5–0·9]); those with low education (1·4 [1·1–1·7]) versus high education (0·8 [0·7–1·0]); low socioeconomic status (1·7 [1·5–2·0]) versus high socioeconomic status (0·9 [0·7–1·1]); and those with (1·6 [1·3–1·9]) versus without depressive symptoms (1·0 [0·9–1·1]; p value range <0·0001–0·0008). Similar differences were seen in women for smoking (2·3 [95% CI 1·9–2·7] *vs* 0·9 [0·7–1·1]), socioeconomic status (1·7 [1·4–2·0] *vs* 0·8 [0·5–1·0]), depressive symptoms (1·4 [1·1–1·7] *vs* 1·0 [0·9–1·1]), and for heavy drinkers compared with moderate drinkers (1·4 [1·1–1·6] *vs* 0·9 [0·7–1·1]; p value range <0·0001–0·010). No differences in physical activity-related health gains were observed between risk groups and non-risk groups by BMI, history of depression, and, in men, alcohol use (p value range 0·11–0·86).

**Interpretation:**

In addition to confirming the association between leisure-time physical activity and increased disease-free years across population subgroups, our findings show that these health benefits are often more pronounced among individuals with pre-existing health risks or disadvantaged backgrounds than in those with more favourable risk factor profiles. This suggests that enhancing population-wide physical activity initiatives could help reduce health disparities, while incorporating physical activity into targeted strategies addressing social disadvantage, unhealthy lifestyles, and depression might enhance their effectiveness.

**Funding:**

Wellcome Trust, UK Medical Research Council, US National Institute on Aging, and Research Council of Finland.

**Research in context**

**Evidence before this study**

We searched PubMed for studies on the association between physical activity and disease-free years, exploring subgroup differences based on socioeconomic status, lifestyle factors, and mental health, without language or date restrictions, from database inception until March 31, 2024. We used the terms “physical activity”, “physical inactivity”, “exercise”, “healthy years”, “healthy life-years”, “disease-free years”, “disease-free life expectancy”, “healthy life expectancy” and “life expectancy”, in addition to reviewing reference lists of relevant publications. We found evidence showing that higher levels of physical activity are associated with a greater number of disease-free years, but the range of diseases included as outcomes varied considerably, and few studies conducted subgroup analyses. Furthermore, we found no large-scale studies that systematically compared the benefits of physical activity on disease-free years between subgroups with high and low risk factor burdens.

**Added value of this study**

To our knowledge, this multicohort study of more than 530 000 initially healthy adults (IPD-Work consortium and UK Biobank) is the first to examine the prospective associations between self-reported leisure-time physical activity and years lived free from major non-communicable diseases across 36 subgroups based on socioeconomic factors (education, socioeconomic status in adulthood), lifestyle (smoking, alcohol consumption, obesity) and mental health (history of depressive disorders, current depressive symptoms). In individuals without coronary heart disease, stroke, type 2 diabetes, cancer, asthma, or chronic obstructive pulmonary disease at baseline, meeting the WHO-recommended levels of physical activity (≥2·5 h of moderate or ≥1·25 h of vigorous physical activity per week) during leisure time was associated with a 1–2-year increment in disease-free life-years after age 40 years, compared with no or very little physical activity. The increase in disease-free years associated with physical activity was consistently observed across all 36 subgroups, in both males and females, and in both studies. However, this increase was generally more pronounced in populations with higher health risks, such as smokers, individuals with lower education or socioeconomic backgrounds, and in those with depressive symptoms.

**Implications of all the available evidence**

In addition to confirming the association between leisure-time physical activity and increased disease-free years across population subgroups, our findings suggest that these health benefits are often greater for individuals with pre-existing health risks or disadvantaged backgrounds than for those with more favourable risk factor profiles. These data suggest that expanding population-wide physical activity initiatives could help reduce health disparities between high-risk and low-risk populations. Furthermore, integrating physical activity into targeted strategies aimed at addressing social deprivation, unhealthy lifestyles, obesity, and depression could enhance the effectiveness of these interventions.

## Introduction

WHO recommends regular physical activity from age 5 years as a key strategy for the primary prevention of non-communicable diseases.^1^ Regular physical activity has been shown to lower the risk of various chronic conditions, including type 2 diabetes, cardiovascular disease, site-specific cancers, and premature death.^2^, ^3^, ^4^ It is also an essential component in alleviating the burden of respiratory diseases, such as chronic obstructive pulmonary disease (COPD),^5^ and in improving disease control and reducing exacerbations in individuals with asthma.^6^ Observational cohort studies further suggest that engaging in regular physical activity during midlife might extend the health span (years of disease-free life).^7^, ^8^, ^9^, ^10^, ^11^, ^12^, ^13^, ^14^, ^15^

Beyond the general population, physical activity can also benefit specific groups. For example, regular exercise has been shown to alleviate symptoms of depression and anxiety.^16^ Moreover, leisure-time physical activity is less prevalent among people with lower socioeconomic status and lifestyle-related risk factors, such as smoking and obesity, and evidence has shown that its potential health benefits in these populations could be substantial.^17^

To date, there is little empirical evidence to determine whether the association of physical activity with chronic disease risk is stronger or weaker in higher risk populations than in individuals with more favourable risk factor profiles. Furthermore, while research shows an association between low physical activity and increased risk of specific chronic diseases, mortality, and life expectancy in various subgroups,^18^, ^19^, ^20^, ^21^, ^22^ the extent to which physical activity might increase years of disease-free life in these groups remains uncertain.^10^ This is a major research gap because the number of disease-free life-years is an important metric for the development of health recommendations and prevention strategies for common chronic diseases and is an easily understandable measure that might help to drive healthy behaviours in the population.

In the present study, we aimed to estimate years of disease-free life in adults with WHO-recommended, intermediate, and low physical activity from leisure time and examine whether the increase in disease-free life-years from higher physical activity differs between 36 subgroups defined by socioeconomical, lifestyle, or mental health factors.

## Methods

### Study design and population

In this multicohort study, our primary analyses used individual-level data from prospective cohort studies within the IPD-Work Consortium: the Finnish Public Sector study (FPS, Finland); Electricité de France-Gaz de France Employees (GAZEL, France); Health and Social Support (HeSSup, Finland); Helsinki Health Study (HHS, Finland); Still Working (Finland); Whitehall II (UK); Work, Lipids, and Fibrinogen Stockholm (WOLF-S, Sweden); and Work, Lipids, and Fibrinogen Norrland Studies (WOLF-N, Sweden). IPD-Work cohort studies were excluded if they had missing outcome data or data restrictions that prevented extraction of individual-level data for pooled analyses. We included participants with no missing data on physical activity who had no prevalent chronic condition at baseline (type 2 diabetes, coronary heart disease, stroke, cancer, asthma, or COPD). The study baseline ranged from March 1, 1986, to Nov 6, 2002, depending on the cohort.

To examine the reproducibility of our findings in an independent population and enhance statistical power for subgroup analyses, we used data from the UK Biobank cohort study, applying the same inclusion criteria as in the primary analysis. Baseline data collection in UK Biobank took place between March 13, 2006, and Oct 1, 2010.

Ethical approval for all studies was obtained from local committees on the ethics of human research and covered the present analyses: FPS: Helsinki Uusimaa Hospital District Ethics Committee (HUS/1210/2016), Finland; GAZEL: The French National Medical Council and the National Consultative Committee of Ethics, (88/25), France; HeSSup: The Turku University Central Hospital Ethics Committee and the Finnish Population Register Centre (VRK2605/410/14); HHS: The ethics committee of the Faculty of Medicine, University of Helsinki (01/2017) and the health authorities of the City of Helsinki, Finland (HEL 2017-004490); Still Working: The Ethics Committee of the Finnish Institute of Occupational Health, Finland; Whitehall II: The University College London Medical School committee on the ethics of human research (85/0938), UK; WOLF-S and WOLF-N N: The Regional Research Ethics Board in Stockholm, and the ethics committee at Karolinska Institutet, Stockholm, Sweden (158-31, 240-32, 92-198, and 03-125). Participants provided informed consent for baseline assessments. Linkage to electronic health records was governed by legislation under consent, or if consent was not required, legitimate interests or Section 251 of the National Health Service Act 2006 and the Health Service (Control of Patient Information) Regulations 2002, depending on the study. Details of the design and participants of each study are described in the appendix (pp 2–4, 7).

### Procedures

In all IPD-Work cohort studies, physical activity was ascertained from participant-completed questionnaires at baseline (appendix pp 2–4). For the main exposure, we used the IPD-Work Consortiums harmonised measure of leisure-time physical activity.^23^ Accordingly, individuals’ physical activity levels were classified as recommended (meeting the WHO recommendation of ≥2·5 h of moderate activity per week or ≥1·25 h of vigorous activity per week), low (no or very little moderate or vigorous physical activity), or intermediate (between these categories).^1^

In all studies, participants were divided into 36 subgroups based on sex, socioeconomic status, lifestyle factors, and mental health status at baseline (appendix pp 5–7, 10–23). Sex data (male *vs* female) were obtained from population registries (FPS, Gazel, HHS, Still Working, WOLF-N, WOLF-S) or was self-reported (HeSSup, Whitehall II). Education was either self-reported or obtained from national registers and was categorised into three levels: low (primary or lower secondary), intermediate (higher secondary), and high (tertiary qualification, college, or university). For IPD-Work cohort studies, adulthood socioeconomic status was determined based on occupational title, which was obtained from employers’ or other registers, or participant-completed questionnaires. In each study, socioeconomic status was categorised as high (eg, professionals or executives), intermediate (eg, skilled non-manual workers), or low (eg, manual workers). In UK Biobank, adulthood socioeconomic status (low, intermediate, high) was defined using the Townsend deprivation index.^24^

Lifestyle risk factors, including BMI, smoking status, and alcohol consumption, were self-reported in most of the studies, except for participants’ height and weight, which were measured in the Whitehall II study, WOLF-N, WOLF-S, and UK Biobank using standard protocols. We calculated BMI as weight in kilograms divided by height in m^2^ and classified participants into categories of healthy weight (BMI 18·5–24·9 kg/m^2^), overweight (BMI 25·0–29·9 kg/m^2^), and obesity (BMI ≥30 kg/m^2^). Smoking status was classified as never, former, or current smoker. Alcohol consumption was categorised as none, moderate (1–14 units per week), or heavy (more than 14 units per week) for both sexes, with sensitivity analyses applying a 21-unit cutoff for heavy consumption in men.

Mental health status was defined as self-reported history with doctor-diagnosed depression (yes or no) and concurrent depressive symptoms (yes or no).

### Outcomes

Details of outcome measurement are provided in the appendix (pp 6–7). Briefly, the primary outcome was disease-free years from a diagnosed major chronic disease, including type 2 diabetes, coronary heart disease, stroke, cancer, asthma, and COPD, as determined from national registry records, clinical examinations, or self-reports. These conditions were selected for their high prevalence and public health significance in high-income countries, prioritisation by WHO for global disease prevention, and their common use in studies of disease-free life-years. In a post-hoc subsidiary analysis, kidney diseases, liver diseases, and dementia were additionally included in the measurement of disease-free years due to their association with low physical activity as a risk factor.

In IPD-Work cohort studies and UK Biobank, participants were prospectively linked to national registries for cancer, hospital admissions, prescription reimbursements, and mortality. In some studies, data were additionally collected from clinical examinations conducted every 5 years or from annual surveys. We excluded participants with a baseline record of any of these chronic diseases or with a record of type 1 diabetes at baseline as they could not develop type 2 diabetes during follow-up.

### Statistical analysis

Briefly, due to sex differences in the age of onset of chronic diseases, analyses were carried out separately for males and females. Disease-free years were defined as the number of life-years between ages 40 years and 75 years during which an individual was free from diagnosis of any of the chronic diseases examined. Age 40 years was chosen as the starting point because it typically marks the onset of health checks, cancer screenings, and cardiovascular risk assessments.

To estimate the associations between self-reported physical activity categories and disease-free years, hazard ratios with 95% CIs for the first disease occurrence were calculated using flexible parametric survival models on the cumulative hazards scale. Restricted cubic splines were fitted within these models to estimate the baseline hazard for each physical activity category using age as the timescale. Each participant contributed data from the time of their physical activity assessment, regardless of their age at that point, until disease onset, death, or end of follow-up, whichever occurred first. Consequently, the full age range was used to estimate survival curves from ages 40 to 75 years.

Disease-free years reached in relation to physical activity categories, compared with the reference group (low physical activity), were calculated as the difference between the areas under the disease-free survival curves from age 40 to 75 years. Area under the curve was computed via numerical integration with a spline-based method. Disease-free years were estimated conditional on survival to age 40 years without any of the chronic diseases investigated. Confidence intervals and p values for disease-free years were estimated via bootstrapping using 1000 independent replications. To test the robustness of the association between physical activity and disease-free life-years in the total study population, we repeated the main analysis using alternative measures of physical activity in sensitivity analyses. For sensitivity analyses, we constructed alternative measures of physical activity, including average metabolic equivalent of task (MET) hours per week and daily television-watching time as a proxy measure for sedentary behaviour. To examine the validity of self-reported measures of physical activity, we assessed the correlation between self-reports and data from wrist-worn accelerometers, which were available post-baseline for subpopulations of FPS, Whitehall II, and UK Biobank participants (details and results are provided in the appendix pp 4, 7–8).

Subgroup analyses stratified by socioeconomic variables were conducted for education (low, intermediate, or high) and adulthood socioeconomic status (low, intermediate, or high). We also stratified analyses by the following lifestyle factors: BMI (healthy weight, overweight, or obese), smoking (current, ex, or never), and alcohol consumption (none, moderate, or heavy drinking). Subgroup analyses by mental health status were based on two variables: history of depression (yes or no) and current depressive symptoms (yes or no). High-risk categories of these subgroup variables included low education, low socioeconomic status, obesity, current smoking, heavy drinking, history of depression, and current depressive symptoms. Participants with missing values in depression, education, socioeconomic status, obesity, smoking, or alcohol consumption were excluded from the respective analyses.

In analysing the IPD-Work cohort studies, we used the maximum pooled data available and included the term ‘cohort study as an additional covariate in the models. We chose this commonly used approach over a two-step meta-analysis, as the latter would have led to data loss due to insufficient numbers in certain subgroups within individual cohorts. To assess the robustness of using pooled individual-level data across all studies for analysing the associations between physical activity and disease-free life-years in the multicohort data and subgroups, we compared results from the pooled analysis with those obtained from a two-stage meta-analysis of cohort-specific estimates. The two-step meta-analytical approach, widely regarded as the gold standard, involves first calculating cohort-specific estimates and then combining these estimates in a meta-analysis. In addition to using a fixed-effects meta-analysis, we also combined the cohort-specific estimates using a random-effects meta-analysis. To examine whether our results were affected by non-proportionality, we repeated the main analysis with time-varying covariates using the tvc option. According to the principles of methodological triangulation, comparable results across these four alternative approaches, each with distinct strengths and limitations, along with an independent replication in the UK Biobank, would support the robustness of our findings.

To test the hypothesis that individuals with health risks or disadvantaged backgrounds derive greater benefits from physical activity than those with more favourable risk factor profiles, we used fixed-effect meta-analysis to combine effect estimates from IPD Work and UK Biobank. We compared physical activity-related gains in disease-free years between high-risk and low-risk groups using *I*^2^ statistics from random-effects meta-analysis.

The prevalence of type 2 diabetes, coronary heart disease, stroke, cancer, asthma, and COPD increases with age, and age can influence physical activity levels. Because participants were required to be free of these diseases at baseline, health-related selection could have affected the results. To explore this possibility, as a post-hoc analysis, we repeated the main analysis in subgroups defined by multiple baseline age groupings (20–40, 30–40, 35–45, 40-50, and 40–60 years). Analyses were conducted using SAS 9.4 and Stata/MP 18.0 statistical software. Further statistical analysis details and the statistical code are provided in the appendix (pp 33–41).

### Role of the funding source

The funders of the study had no role in study design, data collection, data analysis, data interpretation, or writing of the report.

## Results

**Figure 1:**
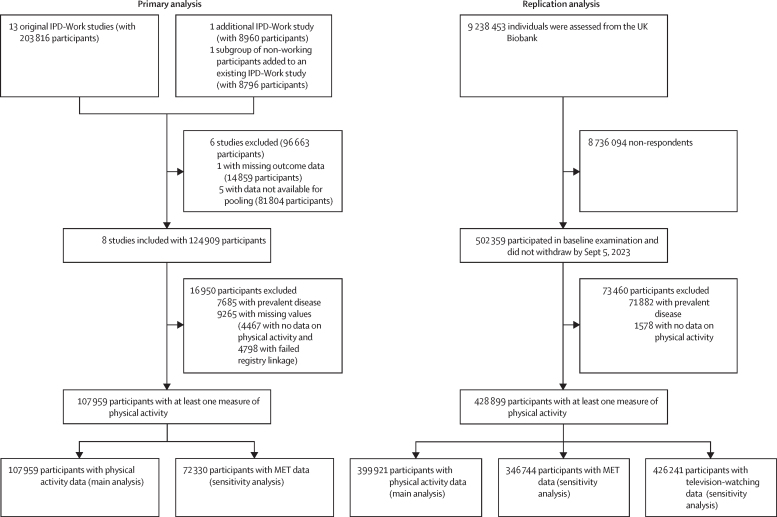
Study selection for the IPD-Work cohorts and UK Biobank MET=metabolic equivalent of task.

14 IPD-Work studies were initially assessed and following the exclusion of six studies due to missing outcome data and data unavailable for pooling, eight studies with 124 909 participants were included. After the exclusion of 7685 participants due to prevalent diseases and 9265 due to missing data, the sample consisted of 107 959 participants with data on at least one measure of physical activity (figure 1). The mean age at baseline of these participants was 43·5 years (SD 10·3), and 63 567 (58·9%) were females and 44 392 (41·1%) were males (table). A total of 23 319 (21·6%) participants were categorised as having low, 32 398 (30·0%) intermediate, and 52 242 (48·4%) recommended physical activity levels during leisure time. During a mean follow-up of 14·5 years, 21 231 incident chronic diseases were recorded.

**Table:** Study designs, participants, and measures of physical activity in IPD-Work cohorts and UK Biobank

	**Baseline year**	**Population**	**Response rate**	**Number of participants (proportion of women)**	**Mean age, years (SD)**	**Measure of physical activity**				**Type of disease follow-up**	**Mean follow-up, years**	**End of follow-up**
						Main survey	MET	Television- watching	Actigraph*			
FPS, Finland	2000	Occupational	68%	44 311 (81%)	44·5 (9·4)	Yes	Yes	No	Yes	Electronic health records	14·5	December, 2016
GAZEL, France	1997	Occupational	75%	9109 (27%)	50·3 (3·0)	Yes	No	No	No	Mortality register; repeated questionnaires	12·4	December, 2010
HeSSup, Finland	1998	General population	40%	21 812 (59%)	36·5 (11·4)	Yes	Yes	No	No	Electronic health records	11·5	December, 2010
HHS, Finland	2000–02	Occupational	67%	6207 (79%)	49·3 (6·6)	Yes	Yes	No	No	Electronic health records	10·9	December, 2012
Still Working, Finland	1986	Occupational	76%	8743 (23%)	40·7 (9·1)	Yes	No	No	No	Electronic health records	24·8	December, 2016
Whitehall II, UK	1991–93	Occupational	73%	8005 (31%)	49·5 (6·1)	Yes	No	No	Yes	Electronic health records; repeated clinical examination; repeated self-report	18·4	December, 2008
WOLF-N, Sweden	1996–98	Occupational	93%	4422 (16%)	43·7 (10·2)	Yes	No	No	No	Electronic health records	11·2	December, 2008
WOLF-S, Sweden	1992–95	Occupational	76%	5350 (42%)	41·2 (11·0)	Yes	No	No	No	Electronic health records	13·9	June, 2015
Pooled dataset	1986–2002	..	62%	107 959 (59%)	43·5 (10·3)	..	..	..	..	..	14·5	..
UK Biobank, UK	2006–10	General population	5%	428 899 (55%)	56·6 (8·1)	Yes	Yes	Yes	Yes	Electronic health records	11·2	November, 2021

MET=metabolic equivalent of task. FPS=Finnish Public Sector study. GAZEL=Electricité de France-Gaz de France Employees. HeSSup=Health and Social Support. HHS=Helsinki Health Study. WOLF-N=Work, Lipids, and Fibrinogen Norrland Studies. WOLF-S=Work, Lipids, and Fibrinogen Stockholm.

* Actigraph measurement available only for a subsample of these studies.

In the UK Biobank, approximately 9 million were individuals invited, 94·5% were non-respondents, and 502 359 participated in the baseline examination. After the exclusion of 73 460 participants (71 882 with prevalent disease and 1578 with no data on physical activity), 428 899 participants had data on at least one measure of physical activity (figure 1). The mean age at baseline was 56·6 years (SD 8·1); 236 258 (55·1%) were female and 192 641 (44·9%) were male. Of 399 921 participants with physical activity data for the main analysis, a total of 182 490 (45·6%) participants were categorised into the low leisure-time physical activity group, 136 774 (34·2%) were categorised as intermediate, and 80 657 (20·2%) were categorised as having recommended physical activity levels. During a mean follow-up of 11·2 years, 101 319 incident chronic diseases were recorded.

**Figure 2:**
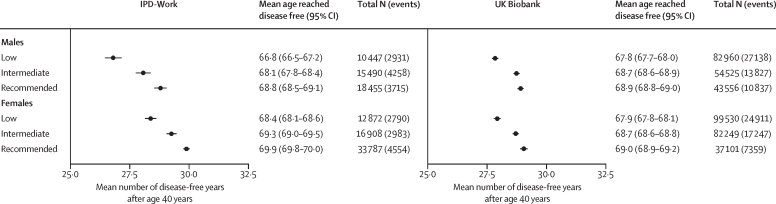
Association of leisure-time physical activity with disease-free years between ages 40 years and 75 years in males and females in the IPD-Work and UK Biobank cohorts Physical activity levels were classified as either low (no or very little moderate or vigorous physical activity), intermediate (<2·5 h of moderate activity per week and <1·25 h of vigorous activity per week) or WHO-recommended (≥2·5 h of moderate activity per week or ≥1·25 h of vigorous activity per week). Diseases included coronary heart disease, stroke, type 2 diabetes, cancer, asthma, and chronic obstructive pulmonary disease. Total N indicates the number of participants at each level of physical activity and events indicate the number of chronic disease cases.

The estimated number of disease-free years was greater for participants reporting higher leisure-time physical activity (figure 2; appendix p 25). In the pooled dataset of IPD-Work cohorts, males with low levels of physical activity had on average 26·8 (95% CI 26·5–27·2) disease-free years after age 40 years. Males with recommended physical activity gained on average 2·0 years (95% CI 1·7–2·3), and those with intermediate physical activity 1·3 years (1·0–1·5) of disease-free life compared with those with a low level of physical activity. In comparison, females with low levels of physical activity had an average of 28·4 (95% CI 28·1–28·6) disease-free years, with those engaging in recommended and intermediate levels of physical activity gaining 1·5 (95% CI 1·3–1·8) and 0·9 (0·6–1·2) additional disease-free years, respectively. The estimates for both sexes were consistent with those obtained from the two-stage fixed-effects and random-effects meta-analyses of cohort-specific data, as well as with those from analyses using the tvc command to account for non-proportionality (appendix pp 33–35).

In UK Biobank, we observed a similar pattern of results, although the effect of leisure-time physical activity was slightly smaller (figure 2, appendix p 25). To account for difference in average baseline age between UK Biobank (mean age 56·6 years) and IPD-Work (43·5 years), we repeated the main analysis in subgroups defined by multiple baseline age groupings. We observed no significant differences in these results across different age groups at the time of physical activity measurement (appendix pp 25–26). For example, the difference in age at which individuals remained disease free between the WHO-recommended and low physical activity groups in UK Biobank participants aged 55 years or younger at baseline (mean age 48·4 years in this subgroup, n=182  703; 1·6 [95% CI 1·3–1·8] in males and 1·5 [1·2–1·7] in females) closely aligns with the findings from the IPD-Work dataset (appendix p 25).

**Figure 3:**
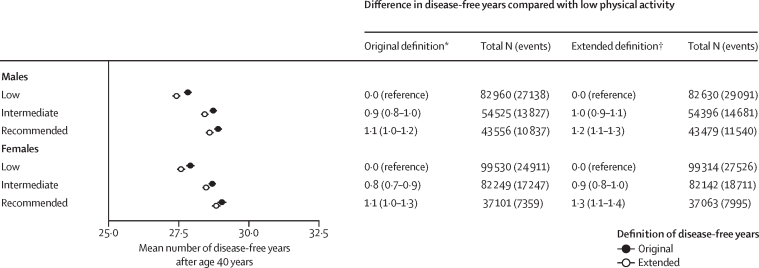
Association between leisure-time physical activity and disease-free years using an extended list of diseases in UK Biobank Physical activity levels were classified as either low (no or very little moderate or vigorous physical activity), intermediate (<2·5 h of moderate activity per week and <1·25 h of vigorous activity per week) or WHO-recommended (≥2·5 h of moderate activity per week or ≥1·25 h of vigorous activity per week). Diseases included coronary heart disease, stroke, type 2 diabetes, cancer, asthma, chronic obstructive pulmonary disease, chronic kidney disease, liver disease, and dementia. *Free of coronary heart disease, stroke, type 2 diabetes, cancer, asthma, or chronic obstructive pulmonary disease. †Additionally free of kidney disease, liver disease, and dementia. Total N indicates the number of participants at each level of physical activity and events indicate the number of chronic disease cases.

In sensitivity analyses using alternative self-reported measures of physical activity, the estimated number of disease-free years was greater for participants with higher physical activity, whether measured by leisure-time physical activities (main exposure), weekly MET hours, or based on a proxy measure of sedentary behaviour (TV-watching time, UK Biobank only; appendix p 27). Furthermore, this trend did not change after including kidney disease, liver disease, and dementia in the measurement of disease-free years (figure 3).

**Figure 4:**
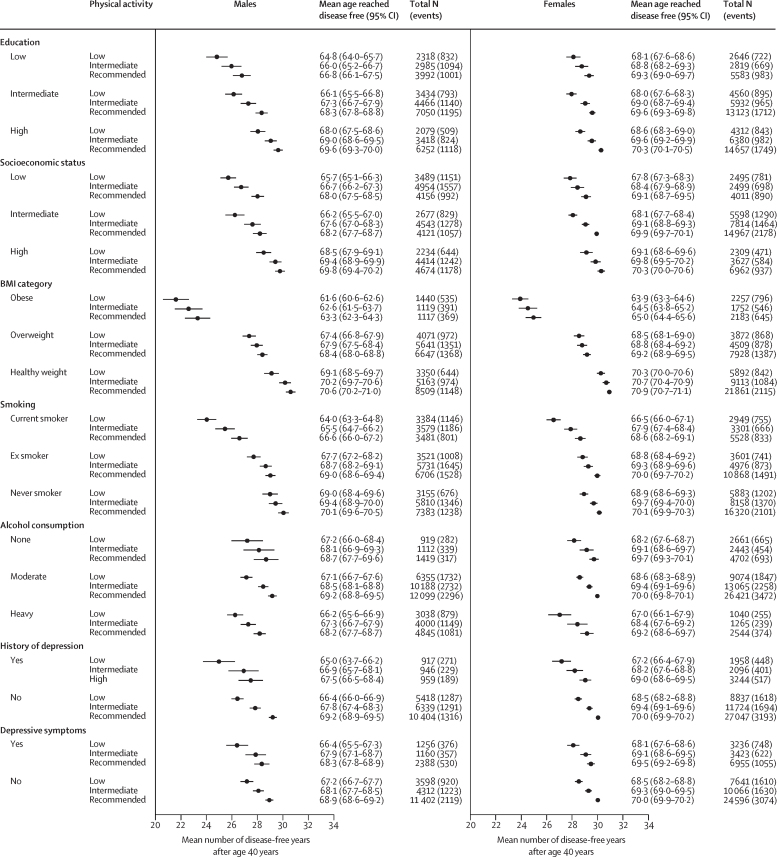
Association of leisure-time physical activity with disease-free years between ages 40 years and 75 years by subgroups in the IPD-Work cohorts Total N indicates the number of participants at each level of physical activity and events indicate the number of chronic disease cases.

Higher leisure-time physical activity in the IPD-Work cohorts was associated with more disease-free years across all 36 subgroups by education level, socioeconomic status, BMI category, smoking status, alcohol consumption, history of depression, and depressive symptoms (figure 4). The health gains from higher physical activity levels in each subgroup were also evident in the UK Biobank study (appendix pp 28–30).

**Figure 5:**
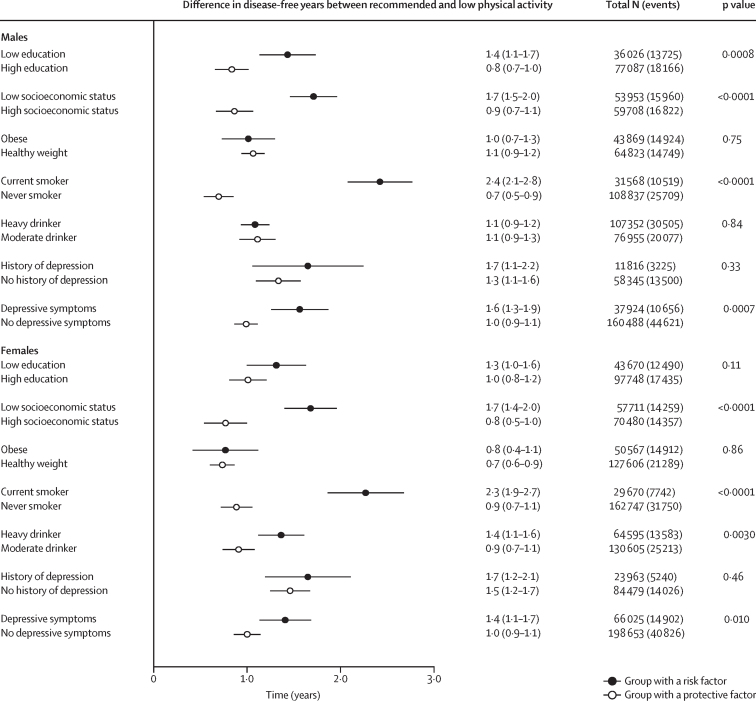
Comparison of disease-free years gained from WHO-recommended leisure-time physical activity between participants in the high-risk versus low-risk categories of socioeconomic factors, lifestyle factors, and depression in the IPD-Work and UK Biobank datasets Total N indicates the number of participants at each level of physical activity and events indicate the number of chronic disease cases.

Meta-analytic pooled estimates from the IPD-Work and UK Biobank studies show that in males, health gains were greater among current smokers than among never smokers, in individuals with low education and low socioeconomic status than among those with higher education and higher socioeconomic status, and in those with depressive symptoms than among those without (p value range <0·0001–0·0008; figure 5; appendix p 32). Similar patterns were observed in females for smoking, socioeconomic status, and depressive symptoms, with an additional difference noted between heavy and moderate drinkers (p value range <0·0001–0·010). No significant differences in physical activity-related health gains were found between risk and non-risk groups by BMI, history of depression, and, in men, alcohol use (p range 0·11–0·86; figure 5).

## Discussion

In this analysis of pooled data from eight IPD-Work cohort studies conducted in Finland, France, Sweden, and the UK, with independent replication in the UK Biobank, we found that recommended levels of physical activity during leisure time are linked to an additional 1–2 years lived without major non-communicable diseases between the ages of 40 years and 75 years. This association persisted regardless of whether the measurement was based on leisure time physical activities, weekly total MET hours (inclusive or exclusive of work, home, and transport-related physical activity), or the use of proxy variables such as TV-watching time. The health benefits of leisure-time physical activity were evident across all 36 subgroups defined by socioeconomic, lifestyle, and mental health factors. However, individuals with high health risks, such as smokers, those with low socioeconomic status, or those with depressive symptoms, gained on average more disease-free years from physical activity than did those with more advantageous backgrounds.

The strengths of this study include the use of a large multinational dataset with harmonised measures and a comprehensive follow-up of chronic diseases using electronic health records. The large size of the study enabled powered analyses within subpopulations. Physical activity mitigates disease risk through multiple mechanisms, including improved blood circulation; enhanced insulin sensitivity and glycaemic control; augmented immune function; reductions in blood pressure and systemic inflammation; and regulation of lipid concentrations, bodyweight, and hormonal balance.^17^ Considering the common causes of chronic diseases across populations, the greater health benefits observed in individuals with pre-existing health risks or disadvantaged backgrounds might reflect their higher burden of risk factors. In these groups, more advanced underlying preclinical pathologies might provide greater scope for the protective effects of physical activity compared with those in more favourable health states.

Although high-risk groups experienced greater benefits from physical activity, their excess disease risks were often not fully eliminated. For example, individuals with obesity who engaged in recommended physical activity had fewer disease-free years after age 40 years compared with their counterparts without obesity but with low physical activity levels. Similarly, current smokers who met recommended physical activity levels did not reach as many disease-free years as never smokers with low physical activity. These findings are consistent with an additive effect of risk and protective factors, aligning with studies indicating a dose–response relationship between a higher number of lifestyle risk factors and a shortened disease-free health span.^13^, ^23^

We are aware of only one previous study, the Finnish Public Sector study, that compared the number of disease-free life-years between different levels of leisure-time physical activity across various subgroups, such as those with high and low socioeconomic status.^10^ This study was part of our pooled IPD-Work database. In agreement with the present findings, the study found that men and women undertaking vigorous physical activity, compared with inactive individuals, lived 2·9 years longer without self-reported diabetes, heart disease, stroke, chronic lung disease, and cancer between ages 50 and 75 years. The corresponding number of years was 3·0 in participants with high socioeconomic status and 2·7 among those with low socioeconomic status.

The present results on the overall health benefits gained from physical activity align broadly with those from other studies. For example, an investigation of four European cohorts reported differences in life-years without chronic diseases between physically active and inactive participants in their leisure time ranging from 1·6 to 4·6 years in men and 1·5 to 3·2 years in women.^7^ In a pooled analysis of data from the US Nurses’ Health Study (n=73 196) and the Health Professionals Follow-Up Study (n=38 366), participants with several low-risk lifestyle factors, including at least 3·5 h per week of moderate to vigorous intensity physical activity, lived longer without type 2 diabetes, cardiovascular disease, and cancer after age 50 years compared with those with a less favourable risk factor profile. The gains from high physical activity were up to 4–6 years, although exact figures were not reported in that study.^13^ Differences in the study populations, the assessment of physical activity, the specific diseases investigated, and the age range at which disease-free years were measured might partially explain the differences in effect estimates between studies, although all of them consistently reported advantages of engaging in physical activity in terms of delaying the onset of non-communicable chronic conditions. Several studies have also quantified the number of extra years associated with physical activity in relation to single diseases and disease groups, such as cardiovascular disease and type 2 diabetes.^8^, ^9^, ^11^

Smokers particularly benefited from being physically active, a pattern previously observed in relation to diseases of the lung.^25^, ^26^ Accordingly, a systematic review and meta-analysis of studies examining the association between physical activity and lung cancer found that the risk reductions associated with physical activity were greater among current smokers than non-smokers.^25^ A similar finding has also been reported in relation to COPD,^26^ and cardiovascular diseases such as stroke.^27^ These findings are plausible for several reasons.^28^ First, physical activity improves cardio-metabolic health via multiple mechanisms,^29^, ^30^ potentially compensating or delaying the cardiovascular damage caused by smoking.^31^ Second, regular exercise has beneficial effects on the respiratory system and, by improving lung function among smokers, it could mitigate the damage caused by smoking-related pathology.^26^, ^32^ Third, physical activity might enhance overall immune function and have an anti-inflammatory influence, thus reducing inflammation resulting from smoking.^33^ Furthermore, physical activity might support smoking cessation, and physically active smokers might smoke fewer cigarettes than physically inactive smokers.^34^, ^35^

Our analyses were based on two large datasets. Despite the harmonisation of the physical activity measure and other measures, some differences in the measurements remained between the studies. Additionally, while the IPD-Work dataset is primarily based on occupational cohort studies with middle-aged participants, our UK Biobank sample included older participants, with only about two-thirds of them being employed. Differences in age distributions, and the potential inclusion of healthier participants in the UK Biobank, might have contributed to variations in the estimated disease-free life-years between the primary and replication datasets. Supporting this, the pattern of results was more similar in a subgroup of UK Biobank participants aged 55 years or younger.

A major limitation of our study is the reliance on self-reported leisure-time physical activity. Self-reports are prone to misclassification, as individuals might have difficulty accurately recalling details of their physical activity, gauging its intensity, or might overestimate their activity levels to present themselves more favourably.^36^ However, our analysis of data from wearable devices revealed an increasing trend in mean acceleration values across the low, intermediate, and recommended levels of self-reported physical activity for both males and females. Although accelerometer measurements captured all daily physical activity, rather than just leisure-time activity, the observed correlation suggests that our questionnaire-based categorisation effectively distinguished between groups with differing average activity levels. Additional limitations of our study include the assessment of disease-free years being primarily based on national registry records of prescription reimbursements, hospital admissions**,** cancer, and mortality**,** which may miss mild cases treated only in primary care, as well as preclinical and undiagnosed conditions. Our study relied on observational data, which precludes causal inference. Furthermore, information on ethnicity was largely unavailable, and many IPD-Work cohorts were limited to employees, restricting the generalisability of our findings. Further large-scale studies are needed to explore the potential benefits of physical activity in more diverse populations.

In conclusion, our findings add to the evidence supporting the WHO recommendation on physical activity as a primary prevention tool for non-communicable diseases in all adults.^1^ Although higher leisure-time physical activity was associated with a longer health span across all 36 subgroups, it appeared to provide greater benefits for individuals with pre-existing health risks or disadvantaged backgrounds, possibly due to a higher prevalence of preclinical or undiagnosed conditions for which physical activity helps to slow further health deterioration. These findings highlight the potential of promoting leisure-time physical activity as a strategy to reduce health disparities between high-risk and low-risk populations.

### Contributors

### Data sharing

Data, protocols, and other metadata for the Whitehall II study are available (Whitehall II study data sharing policy at: https://www.ucl.ac.uk/whitehallII/data-sharing.) Pseudonymised questionnaire data can be requested from the principal investigator by contacting author MZ (marie.zins@inserm.fr), GAZEL; author LA (lars.alfredsson@ki.se), WOLF-N and WOLF-S studies; author JE (jenni.ervasti@ttl.fi), FPS; author SSu (sakari.suominen@utu.fi), HeSSup; author TL (tea.lallukka@helsinki.fi) HHS; and author AV (ari.vaananen@ttl.fi), Still Working. Linked health records for FPS, HeSSuP, HHS, and Still Working require separate permission from the Findata, the Health and Social Data Permit Authority. Researchers registered with UK Biobank can apply for access to the database by completing an application, which must include a summary of the research plan, data fields required, any new data or variables that will be generated, and payment to cover the incremental costs of servicing an application (https://www.ukbiobank.ac.uk/enable-your-research/apply-for-access).

## Declaration of interests

We declare no competing interests.
