# Supplementary material for: Health benefits of leisure-time physical activity by socioeconomic status, lifestyle risk, and mental health: a multicohort study
Source: Lancet Public Health. 2025 Feb 3;10(2):e124–35. doi: 10.1016/S2468-2667(24)00300-1 (PMC11803518; doi:10.1016/S2468-2667(24)00300-1)
Supplement: Supplementary appendix [file mmc1.pdf]

THE LANCET
Public Health

Supplementary appendix

This appendix formed part of the original submission and has been peer reviewed. We post it as supplied by the authors.

Supplement to: Nyberg ST, Frank P, Pentti J, et al. Health benefits of leisure-time physical activity by socioeconomic status, lifestyle risk, and mental health: a multicohort study. *Lancet Public Health* 2025; **10:** e124–35.

Supplementary appendix

Supplement to: Solja T Nyberg, Philipp Frank, Jaana Pentti, Lars Alfredsson, Jenni Ervasti, Marcel Goldberg, Anders Knutsson, Aki Koskinen, Tea Lallukka, Maria Nordin, Ossi Rahkonen, Timo Strandberg, Sakari Suominen, Ari Väänänen, Jussi Vahtera, Marianna Virtanen, Hugo Westerlund, Marie Zins, Sari Stenholm, Severine Sabia, Archana Singh-Manoux, Mark Hamer, Mika Kivimäki: Comparing health benefits of leisure-time physical activity by socioeconomic status, lifestyle risk, and mental health: a multicohort study

List of contents

Description of IPD-Work cohort studies and measurement of physical activity (primary analysis) 2

Assessment of accelerometer-based physical activity 4

Assessment of age, sex, education, socioeconomic status, lifestyle factors, and depression 5

Ascertainment of major non-communicable diseases during follow-up 6

Description of the UK Biobank cohort study (replication analysis) 7

Association between self-reported leisure-time physical activity and accelerometer-based physical activity (validation analysis) 8

Figure S1. Mean (95% CI) level of accelerometer-based physical activity in male and female participants with low, intermediate and recommended self-reported physical activity 8

Table S1. Distribution of physical activity by age group, sex and cohort 9

Table S2. Distribution of physical activity by subgroup and cohort 10

Figure S2. Age distribution in IPD-Work and UK Biobank 24

Figure S3. Association of leisure-time physical activity with disease-free years between ages 40 and 75 in men and women in the IPD-Work and UK Biobank cohorts 25

Figure S4. Association of leisure-time physical activity with disease-free years between ages 40 and 75 in men and women in the UK Biobank cohorts with and without restricting the age at baseline 25

Figure S5. Association of leisure-time physical activity with disease-free years between ages 40 and 75 in men and women in the IPD-Work with and without restricting the age at baseline 26

Figure S6. Association of leisure-time physical activity, MET and daily TV watching time with disease-free years between ages 40 and 75 in men and women in the IPD-Work and UK Biobank cohorts 27

Figure S7. Association of leisure-time physical activity with disease-free years between ages 40 and 75 in men and women in the IPD-Work and UK Biobank cohorts by education and SES 28

Figure S8. Association of leisure-time physical activity with disease-free years between ages 40 and 75 in men and women in the IPD-Work and UK Biobank cohorts by lifestyle categories 29

Figure S9. Association of leisure-time physical activity with disease-free years between ages 40 and 75 in men and women in the IPD-Work and UK Biobank cohorts by mental health status 30

Figure S10. Association of leisure-time physical activity with disease-free years between ages 40 and 75 in men and women in the IPD-Work with different categorisation for alcohol consumption 31

Figure S11. Comparison of disease-free years gained from recommended leisure-time physical activity between participants in the high-risk versus low-risk categories of socioeconomic, lifestyle factors and depression separately in the IPD-Work cohorts and UK Biobank 32

Extended statistical methods 33
Table S3. Comparison of effect estimates between 1-step pooled analysis and 2-step individual-participant meta-analysis 33
Table S4. The number of knots for models selected using the Akaike Information Criteria (AIC) 34
Table S5. Comparison of the effect estimates between the main analysis and a sensitivity analysis allowing for time-dependent effects for physical activity 35
Table S6. The age distribution of participants at the baseline 36
Table S7. Comparison of disease-free years gained from recommended leisure-time physical activity between participants in the high-risk versus low-risk categories of socioeconomic, lifestyle factors and depression in the IPD-Work and UK Biobank datasets 37
Statistical code 39
References 42
STROBE Statement 46

Description of IPD-Work cohort studies and measurement of physical activity (primary analysis)

The IPD-Work cohort studies included in this study were the Finnish Public Sector study (FPS, Finland); Electricité de France-Gaz de France Employees (GAZEL, France); Health and Social Support (HeSSup, Finland); Helsinki Health Study (HHS, Finland); Still Working (Finland); Whitehall II (United Kingdom); Work, Lipids, and Fibrinogen Stockholm (WOLF-S, Sweden); and Work, Lipids, and Fibrinogen Norrland Studies (WOLF-N, Sweden).

Legal basis for European and UK studies in the General Data Protection Regulation (GDPR), the two lawful bases for data processing relating to the legislation as "legitimate interests" and "consent". In the Whitehall II study, an additional legal basis for the linkage to electronic health records is section 251 of the National Health Service Act 2006 and its current Regulations, the Health Service (Control of Patient Information) Regulations 2002.

For the main exposure, we used the IPD-Work Consortiums harmonised measure of leisure-time physical activity, with levels of physical activity classified as either recommended (meeting the Word Health Organization recommendation of ≥2.5 hours of moderate activity per week or ≥1.25 hours of vigorous activity per week),[1] low (no or very little moderate/vigorous physical activity), or intermediate (between the above categories).[2-4]

We focused on leisure-time physical activity rather than occupational physical activity because research indicates that leisure-time physical activity is associated with positive health outcomes, while occupational physical activity tends to have the opposite effect.[5-8]

A summary of physical activity measures in each IPD-Work cohort study is provided in the box, with a more detailed description available below.

Study	Country	Measurement of physical activity (main exposure)	Additional self-reported measures of physical activity*	
			MET	TV
FPS	Finland	The weekly duration of activities by level of intensity, such as walking, brisk walking, jogging, or running, whether during leisure time or commuting, was enquired about using the following response options: 'not at all,' 'less than half an hour,' 'approximately one hour,' '2–3 hours,' and '4 hours or more.' Physical inactivity was defined as engaging in less than 0.5 hour per week of each activity (brisk walking, jogging, or running). The weekly amounts of both moderate and vigorous activities were calculated to classify moderate or high activity levels.	✓	
Gazel	France	Physical activity was enquired with the following response alternatives: Yes, competitively (high), regularly at least once a week (moderate), occasionally (moderate) or no (inactive).		
HeSSup	Finland	The weekly duration of activities by level of intensity, such as walking, brisk walking, jogging, or running, whether during leisure time or commuting, was enquired about using the following response options: 'not at all,' 'less than half an hour,' 'approximately one hour,' '2–3 hours,' and '4 hours or more.' Physical inactivity was defined as engaging in less than 0.5 hour per week of each activity (brisk walking, jogging, or running). The weekly amounts of both moderate and vigorous activities were calculated to classify moderate or high activity levels.	✓	
HHS	Finland	The weekly duration of activities by level of intensity, such as walking, brisk walking, jogging, or running, whether during leisure time or commuting, was enquired about using the following response options: 'not at all,' 'less than half an hour,' 'approximately one hour,' '2–3 hours,' and '4 hours or more.' Physical inactivity was defined as engaging in less than 0.5 hour per week of each activity (brisk walking, jogging, or running). The weekly amounts of both moderate and vigorous activities were calculated to classify moderate or high activity levels.	✓	
Still Working	Finland	"How often do you dedicate your leisure time to physical exercise and sports?" Response options 1 = "daily or almost daily", 2 = "once a week", 3 = "couple of times per month", 4 = "couple of times per year" and 5 = "never". Low (4-5), intermediate (2-3), high (1).		
Whitehall II	UK	Weekly hours spent in moderately energetic or vigorous sports or activities were requested. Physical inactivity was defined as "no moderate or vigorous exercise". Otherwise the weekly amount of both moderate and vigorous activities was used for classifying moderate or high activity.		
WOLF N	Sweden	Physical activity was based on responses from the questionnaire. "No or very little exercise, only occasional walks" was classified as physical inactivity, occasional exercise was classified as moderate and regular exercise as high physical activity.		
WOLF S	Sweden	Physical activity was based on responses from the questionnaire. "No or very little exercise, only occasional walks" was classified as physical inactivity, occasional exercise was classified as moderate and regular exercise as high physical activity.		
**UK Biobank**	UK	Weekly amount of moderate and vigorous physical activities were calculated using frequency and duration of both types of activities.	✓	✓

Box. Measurement of physical activity in IPD-Work and UK Biobank studies
* MET = metabolic equivalent of task; TV = TV watching time

Finnish Public Sector study (FPS), Finland[9]

The Finnish Public Sector study is a prospective cohort study comprising the entire public sector personnel of 10 towns (municipalities) and 21 hospitals in the same geographical areas. Participants, who were recruited from employers' records in 2000-2002, were individuals who had been employed in the study organisations for at least six months prior to data collection. 48 592 individuals (9 337 men and 39 255 women aged 17 to 65) responded to the questionnaire. Ethical approval was obtained from the ethics committee of the Finnish Institute of Occupational Health.

Physical activity was assessed by inquiring about the weekly duration of activities at varying levels of intensity, such as walking, brisk walking, jogging, or running, whether during leisure time or commuting. The response options provided were: 'not at all,' 'less than half an hour,' 'approximately one hour,' '2–3 hours,' and '4 hours or more. Low physical activity was defined as less than 0.5 hour of each (brisk walking, jogging or running) per week. The weekly amount of both moderate and vigorous activities was calculated for classifying intermediate or recommended activity.

Average metabolic equivalent of task (MET) hours per week were based on the average weekly hours of physical activity (including both leisure-time and commuting activity) in walking, brisk walking, jogging, and running, or activities of equivalent intensities, and categorised as low (<14 MET-h/week), high (≥30 MET-h/week) or intermediate (between the two categories).[10]

Gazel, France[11]

Gazel is a prospective cohort study of 20 625 employees (15 011 men and 5 614 women) of France's national gas and electricity company, Electricité de France-Gaz de France (EDF-GDF). Since the study baseline in 1989, when the participants were aged 35–50 years, they have been posted an annual follow-up questionnaire to collect data on health, lifestyle, individual, familial, social, and occupational factors. Gazel in 1997 was treated as a baseline year for our analyses. 11 448 individuals participated that year. The GAZEL study received approval from the national commission overseeing ethical data collection in France (Commission Nationale Informatique et Liberté).

Physical activity was enquired in the questionnaire with the following response alternatives: Yes, competitively (recommended), regularly at least once a week (intermediate), occasionally (intermediate) or no (low)."

Health and Social Support (HeSSup), Finland[12]

The Health and Social Support (HeSSup) study is a prospective cohort study of a stratified random sample of the Finnish population in the following four age groups: 20–24, 30–34, 40–44, and 50–54. The participants were identified from the Finnish population register and posted an invitation to participate, along with a baseline questionnaire, in 1998. 25 898 individuals responded to the questionnaire in 1998. The Turku University Central Hospital Ethics Committee approved the study.

Physical activity was assessed by inquiring about the weekly duration of activities at varying levels of intensity, such as walking, brisk walking, jogging, or running, whether during leisure time or commuting. The response options provided were: 'not at all,' 'less than half an hour,' 'approximately one hour,' '2–3 hours,' and '4 hours or more. Low physical activity was defined as less than 0.5 hour of each (brisk walking, jogging or running) per week. The weekly amount of both moderate and vigorous activities was calculated for classifying intermediate or recommended activity.

Average metabolic equivalent of task (MET) hours per week were based on the average weekly hours of physical activity (including both leisure-time and commuting activity) in walking, brisk walking, jogging, and running, or activities of equivalent intensities, and categorised as low (<14 MET-h/week), high (≥30 MET-h/week) or intermediate (between the two categories).[10]

Helsinki Health Study (HHS), Finland[13]

The Finnish Helsinki Health Study (HHS) is a prospective cohort study comprising all employees of the City of Helsinki, who turned 40, 45, 50, 55, or 60 years in 2000–2002. We included in this study all participants who responded to the baseline survey (n=8960, response rate 67%, 80% women) and provided an informed written consent to combine their survey responses with retrospective and prospective register-based follow-up data on different diseases and mortality (n=6603). Ethical approvals for this study were obtained from the ethics committees of the health authorities of the City of Helsinki, and the Department of Public Health, University of Helsinki.

Physical activity was assessed by inquiring about the weekly duration of activities at varying levels of intensity, such as walking, brisk walking, jogging, or running, whether during leisure time or commuting. The response options provided were: 'not at all,' 'less than half an hour,' 'approximately one hour,' '2–3 hours,' and '4 hours or more. Low physical activity was defined as less than 0.5 hour of each (brisk walking, jogging or running) per week. The weekly amount of both moderate and vigorous activities was calculated for classifying intermediate or recommended activity.

Average metabolic equivalent of task (MET) hours per week were based on the average weekly hours of physical activity (including both leisure-time and commuting activity) in walking, brisk walking, jogging, and running, or activities of equivalent intensities, and categorised as low (<14 MET-h/week), high (≥30 MET-h/week) or intermediate (between the two categories).[10]

Still Working, Finland[14]

Still Working is an ongoing prospective cohort study. In 1986, the employees (n = 12 173) at all Finnish centres of operation of Enso Gutzeit (a forestry products manufacturer) were invited to participate in a questionnaire survey on demographic, psychosocial and health-related factors, and 9 282 individuals participated. The study was approved by the ethics committee of the Finnish Institute of Occupational Health.

Physical activity was ascertained with the questionnaire item inquiring: "How often do you dedicate your leisure time to physical exercise and sports?" Response options 1 = "daily or almost daily", 2 = "once a week", 3 = "couple of times per month", 4 = "couple of times per year" and 5 = "never". Responses were categorised as follows: low (4-5), intermediate (2-3), recommended (1).

Whitehall II, UK[15]

The Whitehall II study is a prospective cohort study set up to investigate socioeconomic determinants of health. At study baseline in 1985-1988, 10 308 civil service employees (6 895 men and 3 413 women) aged 35-55 and working in 20 civil service departments in London were invited to participate in the study. The Whitehall II study protocol was approved by the University College London Medical School committee on the ethics of human research. Written informed consent was obtained at each data collection wave.

Physical activity was defined with the questionnaire items requesting weekly hours spent in moderately energetic or vigorous sports or activities. Low physical activity was defined as “no moderate or vigorous exercise”. Otherwise, the weekly amount of both moderate and vigorous activities was used for classifying intermediate or recommended activity.

WOLF (Work, Lipids, and Fibrinogen) Stockholm and WOLF Norrland studies, Sweden[16,17]

The WOLF (Work, Lipids, and Fibrinogen) Stockholm study is a prospective cohort study of 5 698 people (3 239 men and 2 459 women) aged 19–70 and working in companies in Stockholm county. WOLF Norrland is a prospective cohort of 4 718 participants aged 19-65 working in companies in Jämtland and Västernorrland counties. At study baseline the participants underwent a clinical examination and completed a set of health questionnaires. For WOLF Stockholm, the baseline assessment was undertaken at 20 occupational health units between November 1992 and June 1995 and for WOLF Norrland at 13 occupational health service units in 1996–98. The Regional Research Ethics Board in Stockholm, and the ethics committee at Karolinska Institutet, Stockholm, Sweden approved the study.

Physical activity was based on responses from the questionnaire. “No or very little exercise, only occasional walks” was classified as low physical activity, occasional exercise was classified as intermediate and regular exercise as recommended physical activity.

Assessment of accelerometer-based physical activity

Data on **accelerometer-based physical activity** were available for subsamples of the FPS and Whitehall II studies for validation of the self-reported physical activity measurement in IPD-Work. For this, we used previously reported measures of average 24-hour physical activity.[18,19] In FPS, the sub-population included 835 FPS participants (mean age 62·4, SD 1·1) who have also participated in the Finnish Retirement and Aging Study, an ongoing longitudinal cohort study of older adults in Finland established in 2013.[20] These participants responded to the physical activity questionnaire and wore triaxial ActiGraph wActiSleep-BT accelerometers (ActiGraph, Pensacola, Florida, USA) on their non-dominant wrist over seven consecutive days and six consecutive nights (between September 2014 and February 2018) and had at least 4 valid wear days. Total volume of waking time physical activity was measured as vector magnitude counts per minute (VM CPM).[18]

In Whitehall II, our analysis was based on 3998 participants (mean age 69·3, SD 5·7) who responded to the physical activity questionnaire and underwent a clinical assessment, wore a triaxial accelerometer (GENEActiv Original; Activinsights Ltd, Kimbolton, Cambs, UK, http://www.geneactiv.org/) on their non-dominant wrist for 9 consecutive days at Phase 11 of the study (2012-2013) for at least 4 valid wear days.[21] The measure of Euclidean Norm Minus One (ENMO) was available to quantify the acceleration related to the movements registered and were expressed in milligravity (mg).[19]

Assessment of age, sex, education, socioeconomic status, lifestyle factors, and depression

Age and **sex** were obtained from population registries (FPS, Gazel, HHS, Still Working, WOLF N, WOLF S) or from questionnaires completed by participants (HeSSup, Whitehall II). [22]

Education was self-reported (HeSSuP, HHS, Still Working, Whitehall, WOLF S, WOLF N), or obtained from national registers (FPS). Education was categorised into low (primary or lower secondary), intermediate (higher secondary), and high (tertiary qualification, college, or university) levels. Harmonised three-level education was not available for Gazel.[23]

SES was based on occupational title obtained from employers' or other registers or questionnaires completed by participants and categorised into low, intermediate, or high. Participants who were self-employed or who had missing data on job title were included in the analyses in the "other" category for SES.[22] Data on adulthood SES was not available for HeSSup.

Participants' height and weight were either measured (Whitehall II, WOLF N, WOLF S) or self-reported (FPS, Gazel, HeSSup, HHS). We calculated **BMI** as weight in kg divided by height in m^2. Participants with missing values for height or weight or BMI values less than 15 kg/m2 or more than 50 kg/m2 were excluded, as in our previous analyses. We classified participants according to BMI-values into healthy weight (BMI 18.5-24.9kg/m2), overweight (BMI 25.0-29.9kg/m2), and obesity (BMI ≥30 kg/m2). Participants with BMI<18.5 (underweight) were excluded from the subgroup analysis of BMI categories. Height and weight were not available for the Still Working study.[24,25]

Smoking was based on self-report and categorised as current smoking, never smoking and ex-smoking in all cohorts.[26]

Information on **alcohol consumption** was extracted from questionnaires completed by participants in all studies. Alcohol consumption was based on the total number of units (10 g of ethanol) a participant consumed in a week and categorised according to the UK Chief Medical Officers' guidelines in which heavy drinking was denoted as a weekly consumption exceeding 14 units for men and women[27]; moderate drinking was defined as consuming 1–14 units per week. Non-drinkers were divided into self-reported lifelong abstainers and former drinkers.[28] In a sensitivity analysis alcohol intake was harmonised into the following categories: none, moderate (women: 1-14, men: 1-21 drinks/week), intermediate (women: 15-20, men: 22-27 drinks/week) and heavy (women: >20, men: >27 drinks/week)[29] The study-specific estimates for weekly consumption of alcohol were determined as follows:

FPS: alcohol consumption was based on the reported amounts of beer, wine or other mild alcoholic beverages and hard liquors. For each category, seven pre-defined answer alternatives were given, and weekly consumption was estimated based on the responses.

Gazel: the participant was asked whether or not he consumed wine, beer/cider or aperitifs/digestives during the previous week. For each, the number of days and maximum quantity per day with given response alternatives was asked. Weekly consumption of alcohol was based on the responses.

HeSSup: alcohol consumption was based on the reported amounts of beer, wine or other mild alcoholic beverages and hard liquors. For each category, seven pre-defined answer alternatives were given, and weekly consumption was estimated based on the responses.

HHS: alcohol consumption was based on the reported amounts of beer/cider, wine or other mild alcoholic beverages and hard liquors. For each category, seven pre-defined answer alternatives were given, and weekly consumption was estimated based on the responses.

Still Working: alcohol consumption was assessed by questions on the number of times the respondent used alcohol per week and whether the effect of alcohol use led to any symptoms.

Whitehall II: units of alcohol consumed (spirits, wines, beer) during the last seven days was enquired and weekly consumption was calculated as a sum of the reported amounts.

WOLF N and WOLF S: the frequency and amount of drinking beer / strong beer / wine / strong wine / spirits was requested and weekly alcohol consumption was derived from the responses.

History with depression was available for FPS, Gazel, HeSSup and HHS. In FPS, HeSSup and HHS it was based on a questionnaire item enquiring whether a doctor had ever diagnosed the participant with depression. In Gazel, this was collected from annual responses requesting health problems during the previous 12 months, indicating frequent depressive states (Etats dépressifs fréquents) or depression (deprime).

Depression caseness was available for FPS, HeSSup, HHS and Whitehall II. In FPS and HHS, depression was measured by the self-administered 12-item General Health Questionnaire (GHQ-12)[30]. GHQ-12 consists of 12

questionnaire items inquiring about specific symptoms. Respondents rate the extent to which they are affected by each of the symptoms (1 = not at all, 2 = as much as usual, 3 = slightly more than usual, 4 = much more than usual). In accordance with previous studies, participants with a rating of 3 or 4 in at least four items of the total measure were coded as cases of common mental disorder.[31,32]

In Whitehall II, depression was assessed using the 30-item version of the GHQ. On the basis of previous studies, participants with a total score of five or more were defined as depression cases.[33]

In HeSSup, the Beck Depression Inventory (BDI) was administered to all participants. It consists of 21 items on a four-point scale, individual responses ranging from 0 to 3. Total score ranges from 0 to 63. In accordance with previous studies, a score of at least ten points was used to separate participants with subclinical mild to severe depression from those without depression.[34,35]

Ascertainment of major non-communicable diseases during follow-up

Major chronic diseases included type 2 diabetes, coronary heart disease, stroke, cancer, asthma, and COPD. These diseases were selected for their high prevalence and public health significance in high-income countries, prioritisation by WHO for global disease prevention,[36] and their common use in studies of disease-free life years.[2,24]

Participants were linked to national registers of hospitalisations, prescription reimbursements, cancer and mortality. In Whitehall II, participants additionally attended to 5-yearly clinical examinations. Data from annual surveys during the follow-up were available for the participants of the Gazel study.

Linked records of major chronic diseases covered both baseline and follow-up. The outcome of interest in the present study was the first record of either incident type 2 diabetes, coronary heart disease, stroke, cancer, asthma or COPD. These specific diseases were selected because they are the commonest major non-communicable diseases in developed countries[36,37] and targets prioritised for global disease prevention by the WHO.

Incident type 2 diabetes was defined as the first record of diagnosis corresponding to ICD-10 code E11 (250 in ICD-9 or ICD-8). We collected records from hospital admissions and discharge registers and mortality registers with a mention of diagnosis of type 2 diabetes in any of the diagnosis codes. Additionally, in the Finnish datasets (FPS, HeSSup, HHS and Still working), participants were also defined as an incident type 2 diabetes case the first time they appeared in the nationwide drug reimbursement register as eligible for type 2 diabetes medication.[38] In the Whitehall II study, type 2 diabetes was ascertained by 2-h oral glucose tolerance test administered every 5 years[39] using World Health Organization criteria and complemented by self-reports of diabetes diagnosis and medication.[40] In the Gazel study, non-fatal cases were based on self-report from annual questionnaires.

Coronary heart disease events were identified from hospital discharge and mortality registers, annual self-report questionnaires, or clinical screening using WHO Multinational Monitoring of Trends and Determinants in Cardiovascular Disease (MONICA) Project criteria. We included all non-fatal myocardial infarctions that were recorded as I21–I22 (ICD-10) or 410 (ICD-9) and coronary deaths recorded as I20–I25 (ICD-10) and 410–414 (ICD-9) in any of the diagnose codes.

Incident stroke was defined with hospital and mortality records (I60, I61, I63, I64 in ICD-10; 430, 431, 433, 434, 436 in ICD-9).[22,41] In the Gazel study, non-fatal stroke cases were based on self-report from annual questionnaires.

Cancers, (C00–C97 in ICD-10 or 140-239 in ICD-9), were identified via national cancer, hospital or mortality records, except for Gazel, in which incident cancer events were ascertained from the employer's medical register and by confirming any self-reported cancer diagnosis with the participant's physician.[42]

Severe asthma (J45 or J46 in ICD-10 or 493 in ICD-9) and COPD exacerbations (J41, J42, J43, and J44 in ICD-10, or 491, 492, and 496 in ICD-9) were ascertained from hospital discharge and death registers in all studies except for Gazel, in which non-fatal asthma events were based on self-report from annual questionnaires and non-fatal COPD was not available.[43,44]

Participants with missing data on these outcomes and those with a record of these diseases already at baseline were excluded from the analyses. We also excluded participants with a record of type 1 diabetes at baseline: E10 (ICD-10) or 250 (ICD-9 and ICD-8).[45]

Description of the UK Biobank cohort study (replication analysis)

The UK Biobank is an ongoing prospective cohort study.[46] Approximately 9.2 million invitations were mailed and during 2006-2010, over half a million men and women aged 40-69 years from the United Kingdom participated. The baseline data collection involved questionnaire and physical measurements. Our analysis was done under a generic approval from the National Health Service National Research Ethics Service (11/NW/0382). The present study was conducted using the UK Biobank Resource under Application Number 60565.

Physical activity was defined by calculating weekly amount of moderate and vigorous physical activities using frequency and duration of both types of activities.

Secondary exposures included MET and TV-watching time. MET was based on the predefined, original version of MET variable that additionally included work- and transport-related physical activity, domestic and gardening activities.[47] We also used a proxy measure of sedentary behaviour, daily TV-watching time, which was categorised into three categories: less than two hours, at least two, but less than four hours and at least four hours per day.

Measures at baseline

In the UK Biobank, sex and age of the participants were obtained from registries. This information could be amended by the participant upon arrival at the Assessment Centre. SES was based on Townsend deprivation index at recruitment.[48] Education was based on self-report. **Height** and **weight** of the participants were measured, and **BMI** was defined similarly as for the main analysis. **Smoking** was based on self-report and categorised as current smoking, never smoking and ex-smoking. Information on **alcohol use** was extracted from questionnaires completed by participants and included the frequency and amount of drinking red wine, champagne / white wine, beer / cider, spirits, fortified wine and other (such as alcopops). Weekly alcohol consumption was derived from the responses and categorised similarly as for the main analysis. **History with depression** was based on predefined derived fields of lifetime major depression (MD) status defined from the touchscreen questionnaire at baseline and included "single episode of probable MD", "probable recurrent MD (moderate)", and "probable recurrent MD (severe)" as history with depression.[49,50] **Depression caseness** was defined present, if the response to baseline question "Frequency of depressed mood in last 2 weeks" was several days or more (vs. not at all).[51]

Accelerometer-based physical activity

Data on **accelerometer-based physical activity** included 93 055 participants (mean age 56.1 years, (SD 7.8).) who responded to the physical activity questionnaire and wore an Axivity AX3 accelerometer (Axivity, Newcastle, UK) on their dominant wrist for 24 h per day for 7 days between June 1, 2013, and Dec 23, 2015. As previously, accelerometer measured physical activity was quantified in milligravity units (mg).[52]

Ascertainment of major non-communicable diseases during follow-up

In UK Biobank, study participants were linked to the UK National Health Service's Hospital Episode Statistics (HES) database for hospital admissions and the NHS Central Registry for mortality from 18-Mar-1995 to 31-Nov-2021, and using this information, major non-communicable diseases during follow-up were defined with a similar manner as for the main analysis.

In subsidiary analyses, chronic kidney disease, end-stage renal disease, fatty liver, cirrhosis, liver failure, and dementia were included due to the association of low physical activity as a risk factor for these conditions.[53-55] ICD-10 diagnostic codes for these age-related conditions, previously used in UK Biobank studies,[56] were based on the recommendations of Fraser et al:[57] N18 (chronic kidney disease), N18.0, N18.5, T82.4, Y60.2, Y61.2, Y84.1, Z49.1, Z49.2, Z99.2, N16.5, T86.1, Z94.0 (end-stage renal disease), K70.0, K75.8, K76.0 (fatty liver), K70.1, K70.2, K70.3, K71.7, K74.0, K74.1, K74.2, K74.4, K74.5, K74.6 (cirrhosis), K70.4, K72 (liver failure), F00, F01, F03, F05.1, G30 (dementia).

Association between self-reported leisure-time physical activity and accelerometer-based physical activity (validation analysis)

The cross-sectional association between self-reported physical activity and accelerometer-based amount of physical activity for a total of 97 779 participants with both sets of data showed a statistically significant increasing trend in mean acceleration values across the low, intermediate, and recommended levels of self-reported physical activity in men and women. These findings support the validity of our questionnaire-based definition of leisure-time physical activity level.

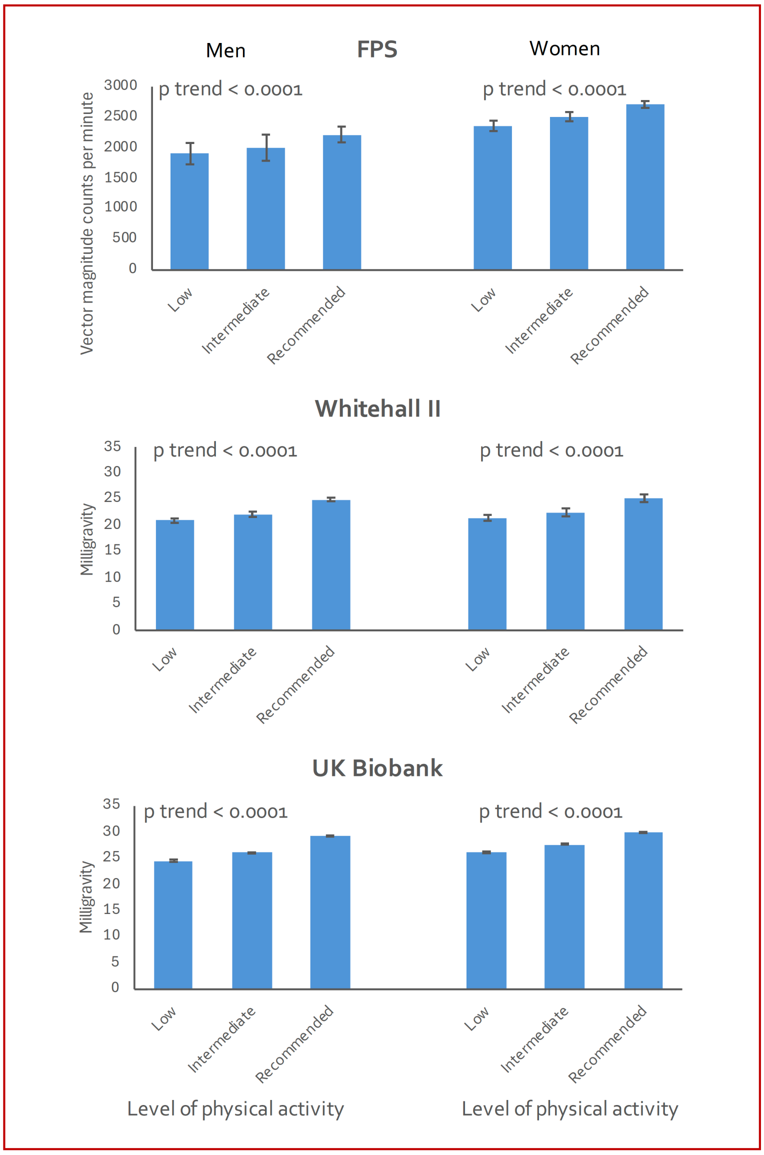

Figure S1. Mean (95% CI) level of accelerometer-based physical activity in male and female participants with low, intermediate and recommended self-reported physical activity
Bars represent vector magnitude per minute in FPS (N = 835), mean acceleration in milligravity in Whitehall II (N = 3998) and average acceleration in UK Biobank (N = 93 055).

Table S1. Distribution of physical activity by age group, sex and cohort

Age	Physical activity				Percentage by level of physical activity			
**IPD-Work, men**	**Low**	**Intermediate**	**Recommended**	**Total**				
<40	2627	3681	6582	12890	20.4	28.6	51.1	100.0
40-49	3390	5535	6103	15028	22.6	36.8	40.6	100.0
50-59	4173	5901	5278	15352	27.2	38.4	34.4	100.0
60+	257	373	492	1122	22.9	33.2	43.9	100.0
Total	10447	15490	18455	44392				
**IPD-Work, women**								
<40	3038	4773	11934	19745	15.4	24.2	60.4	100.0
40-49	4476	6333	11496	22305	20.1	28.4	51.5	100.0
50-59	4889	5366	9627	19882	24.6	27.0	48.4	100.0
60+	469	436	730	1635	28.7	26.7	44.6	100.0
Total	12872	16908	33787	63567				

…continues

UK Biobank, men	Physical activity							
	**Low**	**Intermediate**	**Recommended**	**Total**	Percentage			
<40	<5	<5	<5	<5				
40-49	14602	15700	12817	43119	33.9	36.4	29.7	100.0
50-59	26123	18219	13919	58261	44.8	31.3	23.9	100.0
60+	42234	20606	16819	79659	53.0	25.9	21.1	100.0
**UK Biobank, women**								
<40	<5	<5	<5	<5				
40-49	18926	21730	10166	50822	37.2	42.8	20.0	100.0
50-59	33944	28602	12162	74708	45.4	38.3	16.3	100.0
60+	46660	31917	14773	93350	50.0	34.2	15.8	100.0

Table S2. Distribution of physical activity by subgroup and cohort

	Physical activity			
**FPS**				
	**low**	**intermediate**	**recommended**	**Total**
**men**	1825	1663	4961	
	21.6	19.68	58.72	8449
**women**	6738	8497	20627	
	18.79	23.69	57.52	35862
**Total**	8563	10160	25588	44311
**Gazel**				**Total**
**men**	**low**	**intermediate**	**recommended**	
	35.06	58.35	6.59	6620
**women**	1041	1402	46	
	41.82	56.33	1.85	2489
**Total**	3362	5265	482	9109
**HeSSup**				
	**low**	**intermediate**	**recommended**	**Total**
**men**	1993	1533	5385	
	22.37	17.2	60.43	8911
**women**	2396	2868	7637	
	18.57	22.23	59.2	12901
**Total**	4389	4401	13022	21812
**HHS**				
	**low**	**intermediate**	**recommended**	**Total**
**men**	290	283	747	
	21.97	21.44	56.59	1320
**women**	921	1348	2618	
	18.85	27.58	53.57	4887
**Total**	1211	1631	3365	6207
**Still working**				
	**low**	**intermediate**	**recommended**	**Total**
**men**	1431	3399	1934	
	21.16	50.25	28.59	6764
**women**	285	964	730	
	14.4	48.71	36.89	1979
**Total**	1716	4363	2664	8743

WOLF N

	low	intermediate	recommended	Total
**men**	1052	1602	1060	
	28.33	43.13	28.54	3714
**women**	141	235	332	
	19.92	33.19	46.89	708
**Total**	1193	1837	1392	4422

WOLF S

	low	intermediate	recommended	Total
**men**	759	1130	1196	
	24.6	36.63	38.77	3085
**women**	475	780	1010	
	20.97	34.44	44.59	2265
**Total**	1234	1910	2206	5350

Whitehall

	low	intermediate	recommended	Total
**men**	776	2017	2736	
	14.04	36.48	49.48	5529
**women**	875	814	787	
	35.34	32.88	31.79	2476
**Total**	1651	2831	3523	8005

Education

FPS

	Low	Intermediate	High	Total
**men**	1157	2760	4532	
	13.69	32.67	53.64	8449
**women**	3737	12461	19663	
	10.42	34.75	54.83	35861
**Total**	4894	15221	24195	44310

Frequency Missing = 1

Gazel / not available

HeSSup	Low	Intermediate	High	Total

**men**	2777	4889	1189	
	31.36	55.21	13.43	8855
**women**	4094	6886	1749	
	32.16	54.1	13.74	12729
**Total**	6871	11775	2938	21584
	**Frequency Missing = 228**			

HHS

	**Low**	**Intermediate**	**High**	**Total**
**men**	251	614	450	
	19.09	46.69	34.22	1315
**women**	929	2655	1267	
	19.15	54.73	26.12	4851
**Total**	1180	3269	1717	6166
	**Frequency Missing = 41**			

Still working

	**Low**	**Intermediate**	**High**	**Total**
**men**	2618	2939	879	
	40.68	45.67	13.66	6436
**women**	911	539	322	
	51.41	30.42	18.17	1772
**Total**	3529	3478	1201	8208
	**Frequency Missing = 535**			

WOLF N

	**Low**	**Intermediate**	**High**	**Total**
**men**	861	1781	1062	
	23.25	48.08	28.67	3704
**women**	151	204	352	
	21.36	28.85	49.79	707
**Total**	1012	1985	1414	4411
	**Frequency Missing = 11**			

WOLF S

	**Low**	**Intermediate**	**High**	**Total**
**men**	525	881	1674	
	17.05	28.6	54.35	3080
**women**	371	449	1444	
	16.39	19.83	63.78	2264
**Total**	896	1330	3118	5344
	**Frequency Missing = 6**			

Whitehall

	Low	Intermediate	High	Total
**men**	1106	1086	1963	
	26.62	26.14	47.24	4155
**women**	855	421	552	
	46.77	23.03	30.2	1828
**Total**	1961	1507	2515	5983

SES

FPS

	Low	Intermediate	High	4	Total
**men**	2742	2228	3350	129	
	32.45	26.37	39.65	1.53	8449
**women**	5288	20813	9433	328	
	14.75	58.04	26.3	0.91	35862
**Total**	8030	23041	12783	457	44311

Gazel

	Low	Intermediate	High	4	Total
**men**	578	3090	2946	6	
	8.73	46.68	44.5	0.09	6620
**women**	404	1730	354	1	
	16.23	69.51	14.22	0.04	2489
**Total**	982	4820	3300	7	9109

HeSSup / not available

HHS

	Low	Intermediate	High	4	Total
**men**	339	121	860	0	
	25.68	9.17	65.15	0	1320
**women**	625	1889	2362	11	
	12.79	38.65	48.33	0.23	4887
**Total**	964	2010	3222	11	6207

Still working

	Low	Intermediate	High	4	Total
**men**	4929	1246	589	0	
	72.87	18.42	8.71	0	6764

**women**	1118	826	35	0	
	56.49	41.74	1.77	0	1979
**Total**	6047	2072	624	0	8743

WOLF N

	**Low**	**Intermediate**	**High**	**4**	**Total**
**men**	2436	968	306	4	
	65.59	26.06	8.24	0.11	3714
**women**	204	459	44	1	
	28.81	64.83	6.21	0.14	708
**Total**	2640	1427	350	5	4422

WOLF S

	**Low**	**Intermediate**	**High**	**4**	**Total**
**men**	1223	1201	587	74	
	39.64	38.93	19.03	2.4	3085
**women**	409	1538	276	42	
	18.06	67.9	12.19	1.85	2265
**Total**	1632	2739	863	116	5350

Whitehall

	**Low**	**Intermediate**	**High**	**4**	**Total**
**men**	352	2487	2684	0	
	6.37	45.03	48.6	0	5523
**women**	957	1124	394	0	
	38.67	45.41	15.92	0	2475
**Total**	1309	3611	3078	0	7998

Frequency Missing = 7

BMI category

FPS

	**BMI <18.5**	**18.5-24.9**	**25-29.9**	**30-34.9**	**35-39.9**	**40->**	**Total**
**men**	15	3528	3833	826	150	34	
	0.18	42.07	45.71	9.85	1.79	0.41	8386
**women**	524	21034	9901	2920	616	159	
	1.49	59.83	28.16	8.31	1.75	0.45	35154
**Total**	539	24562	13734	3746	766	193	43540

Frequency Missing = 771

Gazel

	**BMI <18.5**	**18.5-24.9**	**25-29.9**	**30-34.9**	**35-39.9**	**40->**	**Total**
**men**	12	2564	3409	536	36	7	6564

	0.18	39.06	51.93	8.17	0.55	0.11	
**women**	71	1751	484	127	26	<5	
	2.88	71.09	19.65	5.16	1.06		
**Total**	83	4315	3893	663	62		

Frequency Missing = 82

HeSSup

	**BMI <18.5**	**18.5-24.9**	**25-29.9**	**30-34.9**	**35-39.9**	**40->**	**Total**
**men**	78	4637	3334	670	131	29	
	0.88	52.22	37.55	7.55	1.48	0.33	8879
**women**	434	8383	2920	841	190	49	
	3.39	65.41	22.78	6.56	1.48	0.38	12817
**Total**	512	13020	6254	1511	321	78	21696

Frequency Missing = 116

HHS

	**BMI <18.5**	**18.5-24.9**	**25-29.9**	**30-34.9**	**35-39.9**	**40->**	**Total**
**men**	<5	517	608	142	35	5	
		39.44	46.38	10.83	2.67	0.38	
**women**	56	2605	1526	517	121	21	
	1.16	53.76	31.49	10.67	2.5	0.43	4846
**Total**		3122	2134	659	156	26	6157

Frequency Missing = 50

Still working / not available

WOLF N

	**BMI <18.5**	**18.5-24.9**	**25-29.9**	**30-34.9**	**35-39.9**	**40->**	**Total**
**men**	9	1414	1815	397	68	9	
	0.24	38.09	48.9	10.7	1.83	0.24	3712
**women**	<5	388	214	77	14	5	
		55.27	30.48	10.97	1.99	0.71	
**Total**		1802	2029	474	82	14	4414

Frequency Missing = 8

WOLF S

	**BMI <18.5**	**18.5-24.9**	**25-29.9**	**30-34.9**	**35-39.9**	**40->**	**Total**
**men**	16	1581	1251	200	30	<5	
	0.52	51.31	40.6	6.49	0.97		
**women**	62	1538	490	130	20	<5	
	2.76	68.51	21.83	5.79	0.89		
**Total**	78	3119	1741	330	50		

Frequency Missing = 24

Whitehall

	BMI <18.5	18.5-24.9	25-29.9	30-34.9	35-39.9	40->	Total
**men**	33	2781	2109	330	33	5	
	0.62	52.56	39.86	6.24	0.62	0.09	5291
**women**	43	1167	774	243	73	34	
	1.84	50	33.16	10.41	3.13	1.46	2334
**Total**	76	3948	2883	573	106	39	7625

Frequency Missing = 380

Smoking

FPS

	never	ex smoker	current smoker	Total
**men**	2616	3620	1918	
	32.08	44.4	23.52	8154
**women**	16139	13038	5751	
	46.21	37.33	16.47	34928
**Total**	18755	16658	7669	43082

Frequency Missing = 1229

Gazel

	never	ex smoker	current smoker	Total
**men**	2380	2991	1249	
	35.95	45.18	18.87	6620
**women**	1605	524	360	
	64.48	21.05	14.46	2489
**Total**	3985	3515	1609	9109

HeSSup

	never	ex smoker	current smoker	Total
**men**	2969	2498	2540	
	37.08	31.2	31.72	8007
**women**	6120	2994	2934	
	50.8	24.85	24.35	12048
**Total**	9089	5492	5474	20055

Frequency Missing = 1757

HHS

	never	ex smoker	current smoker	Total
**men**	536	441	336	
	40.82	33.59	25.59	1313
**women**	2742	1026	1085	4853

	56.5	21.14	22.36	
**Total**	3278	1467	1421	6166
	**Frequency Missing = 41**			

**Still working**	**never**	**ex smoker**	**current smoker**	**Total**
**men**	1988	2324	2410	
	29.57	34.57	35.85	6722
**women**	1067	436	464	
	54.25	22.17	23.59	1967
**Total**	3055	2760	2874	8689
	**Frequency Missing = 54**			

**WOLF N**	**never**	**ex smoker**	**current smoker**	**Total**
**men**	1817	1121	664	
	50.44	31.12	18.43	3602
**women**	319	200	164	
	46.71	29.28	24.01	683
**Total**	2136	1321	828	4285
	**Frequency Missing = 137**			

**WOLF S**	**never**	**ex smoker**	**current smoker**	**Total**
**men**	1414	879	725	
	46.85	29.13	24.02	3018
**women**	1027	587	615	
	46.07	26.33	27.59	2229
**Total**	2441	1466	1340	5247
	**Frequency Missing = 103**			

**Whitehall**				
**men**	2628	2084	602	
	49.45	39.22	11.33	5314
**women**	1342	640	405	
	56.22	26.81	16.97	2387
**Total**	3970	2724	1007	7701

Alcohol consumption

**FPS**	**None**	**Moderate**	**High**	**Total**

**male**	612	5093	2702	
	7.28	60.58	32.14	8407
**female**	5369	27336	3016	
	15.03	76.53	8.44	35721
**Total**	5981	32429	5718	44128
	**Frequency Missing = 183**			

Gazel

	**None**	**Moderate**	**High**	**Total**
**male**	477	3667	2331	
	7.37	56.63	36	6475
**female**	574	1637	190	
	23.91	68.18	7.91	2401
**Total**	1051	5304	2521	8876
	**Frequency Missing = 233**			

HeSSup

	**Non**	**Moderate**	**High**	**Total**
**male**	886	5596	2418	
	9.96	62.88	27.17	8900
**female**	2513	9345	1028	
	19.5	72.52	7.98	12886
**Total**	3399	14941	3446	21786
	**Frequency Missing = 26**			

HHS

	**None**	**Moderate**	**High**	**Total**
**male**	74	998	245	
	5.62	75.78	18.6	1317
**female**	328	4389	144	
	6.75	90.29	2.96	4861
**Total**	402	5387	389	6178
	**Frequency Missing = 29**			

Still working

	**None**	**Moderate**	**High**	**Total**
**male**	273	5102	1333	
	4.07	76.06	19.87	6708
**female**	140	1717	105	
	7.14	87.51	5.35	1962
**Total**	413	6819	1438	8670
	**Frequency Missing = 73**			

WOLF N

	Non	Moderate	High	Total
**male**	223	2882	533	
	6.13	79.22	14.65	3638
**female**	49	626	15	
	7.1	90.72	2.17	690
**Total**	272	3508	548	4328

Frequency Missing = 94

WOLF S

	None	Moderate	High	Total
**male**	110	2282	615	
	3.66	75.89	20.45	3007
**female**	88	2008	124	
	3.96	90.45	5.59	2220
**Total**	198	4290	739	5227

Frequency Missing = 123

Whitehall

	None	Moderate	High	Total
**male**	795	3022	1706	
	14.39	54.72	30.89	5523
**female**	745	1502	227	
	30.11	60.71	9.18	2474
**Total**	1540	4524	1933	7997

Frequency Missing = 8

History with depression

FPS

	0	1	Total
**men**	7475	811	
	90.21	9.79	8286
**women**	31103	4072	
	88.42	11.58	35175
**Total**	38578	4883	43461

Frequency Missing = 850

Gazel

	0	1	Total
**men**	5577	1043	
	84.24	15.76	6620
**women**	1474	1015	2489

	59.22	40.78	
**Total**	7051	2058	9109

HeSSup

	**0**	**1**	**Total**
**men**	7995	850	
	90.39	9.61	8845
**women**	11231	1598	
	87.54	12.46	12829
**Total**	19226	2448	21674
	**Frequency Missing = 138**		

HHS

	**0**	**1**	**Total**
**men**	1114	118	
	90.42	9.58	1232
**women**	3800	613	
	86.11	13.89	4413
**Total**	4914	731	5645
	**Frequency Missing = 562**		

Still working / not available

WOLF N / not available

WOLF S / not available

Whitehall / not available

Depressive symptoms

FPS

Frequency

	**0**	**1**	**Total**
**men**	6458	1945	
	76.85	23.15	8403
**women**	26401	9297	
	73.96	26.04	35698
**Total**	32859	11242	44101
	**Frequency Missing = 210**		

Gazel / not available

HeSSup

	**0**	**1**	**Total**

**men**	7409	1473	
	83.42	16.58	8882
**women**	10181	2705	
	79.01	20.99	12886
**Total**	17590	4178	21768

Frequency Missing = 44

HHS

	**0**	**1**	**Total**
**men**	1064	245	
	81.28	18.72	1309
**women**	3874	987	
	79.7	20.3	4861
**Total**	4938	1232	6170

Frequency Missing = 37

Still working / not available
WOLF N / not available
WOLF S / not available

Whitehall

	**0**	**1**	**Total**
**men**	4381	1141	
	79.34	20.66	5522
**women**	1847	625	
	74.72	25.28	2472
**Total**	6228	1766	7994

Frequency Missing = 11

UK Biobank

Physical activity

	**Low**	**Intermediate**	**Recommended**	**Total**
**Men**	82960	54525	43556	
	45.82	30.12	24.06	181041
**Women**	99530	82249	37101	
	45.47	37.58	16.95	218880
**Total**	182490	136774	80657	399921

Frequency Missing = 28978

Education

Frequency

	**Low**	**Intermediate**	**High**	**Total**
**Men**	26731	87260	65338	
	14.91	48.66	36.43	179329
**Women**	32622	111935	72399	
	15.04	51.59	33.37	216956
**Total**	59353	199195	137737	396285

Frequency Missing = 3636

SES

Frequency

	**High**	**Intermediate**	**Low**	**Total**
**Men**	48386	91069	41354	
	26.76	50.37	22.87	180809
**Women**	57582	112344	48706	
	26.34	51.38	22.28	218632
**Total**	105968	203413	90060	399441

Frequency Missing = 480

	**BMI <18.5**	**18.5-24.9**	**25-29.9**	**30-34.9**	**35-39.9**	**40->**	**Total**
**Men**	375	47801	91839	32628	6183	1382	
	0.21	26.53	50.96	18.11	3.43	0.77	180208
**Women**	1659	90740	81161	31283	9721	3371	
	0.76	41.64	37.24	14.35	4.46	1.55	217935
**Total**	2034	138541	173000	63911	15904	4753	398143

Frequency Missing = 1778

**Frequency**	**Never smoker**	**Ex smoker**	**Current smoker**	**Total**
**Men**	92489	66822	21124	
	51.26	37.03	11.71	180435
**Women**	132386	67908	17892	
	60.68	31.12	8.2	218186
**Total**	224875	134730	39016	398621

Frequency Missing = 1300

History with depression

Frequency			
	**No**	**Yes**	**Total**
**Men**	36184	8994	
	80.09	19.91	45178
**Women**	36871	16665	
	68.87	31.13	53536
**Total**	73055	25659	98714

Frequency Missing = 301207

Depressive symptoms

	**No**	**Yes**	**Total**
**Men**	141176	33120	
	81	19	174296
**Women**	156350	52411	
	74.89	25.11	208761
**Total**	297526	85531	383057

Frequency Missing = 16864

Alcohol consumption

Frequency				
	**None**	**Moderate**	**High**	**Total**
**Men**	36687	48313	95469	
	20.33	26.77	52.9	180469
**Women**	75955	82045	59746	
	34.88	37.68	27.44	217746
**Total**	112642	130358	155215	398215

Frequency Missing = 1706

Figure S2. Age distribution in IPD-Work and UK Biobank

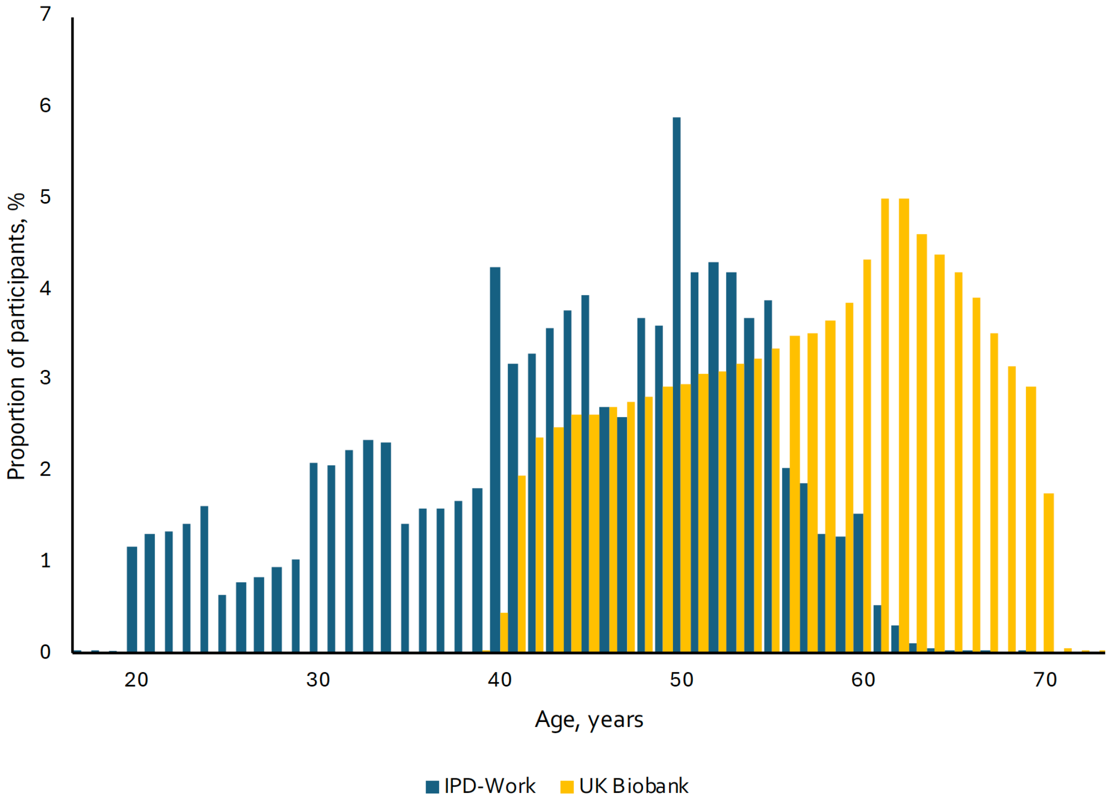

Figure S3. Association of leisure-time physical activity with disease-free years between ages 40 and 75 in men and women in the IPD-Work and UK Biobank cohorts

Physical activity	Total N (events)	IPD-Work	Difference (95% CI)	Total N (events)	UK Biobank	Difference (95% CI)
**Men**						
Low	10 447 (2931)		0.0 (reference)	82 960 (27 138)		0.0 (reference)
Intermediate	15 490 (4258)		1.3 (1.0 to 1.5)	54 525 (13 827)		0.9 (0.8 to 1.0)
Recommended	18 455 (3715)		2.0 (1.7 to 2.3)	43 556 (10 837)		1.1 (1.0 to 1.2)
**Women**						
Low	12 872 (2790)		0.0 (reference)	99 530 (24 911)		0.0 (reference)
Intermediate	16 908 (2983)		0.9 (0.6 to 1.2)	82 249 (17 247)		0.8 (0.7 to 0.9)
Recommended	33787 (4554)		1.5 (1.3 to 1.8)	37 101 (7359)		1.1 (1.0 to 1.3)

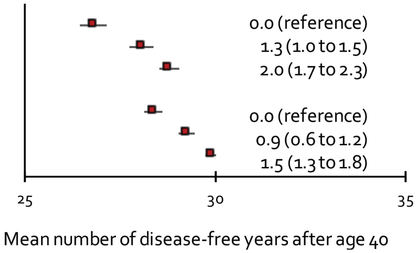

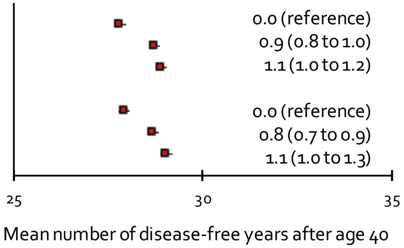

Figure S4. Association of leisure-time physical activity with disease-free years between ages 40 and 75 in men and women in the UK Biobank cohorts with and without restricting the age at baseline

	UK Biobank men		UK Biobank women	
Physical activity	Total N (events)	Mean age reached disease free (95% CI)	Total N (events)	Mean age reached disease free (95% CI)
**Total sample**				
Low		67.8 (67.7 to 68.0)		67.9 (67.8 to 68.1)
Intermediate		68.7 (68.6 to 68.9)		68.7 (68.6 to 68.8)
Recommended		68.9 (68.8 to 69.0)		69.0 (68.9 to 69.2)
**Baseline age ≤55 years**				
Low		67.7 (67.5 to 67.8)		67.9 (67.7 to 68.0)
Intermediate		68.9 (68.7 to 69.1)		68.9 (68.8 to 69.1)
Recommended		69.3 (69.1 to 69.4)		69.4 (69.2 to 69.6)

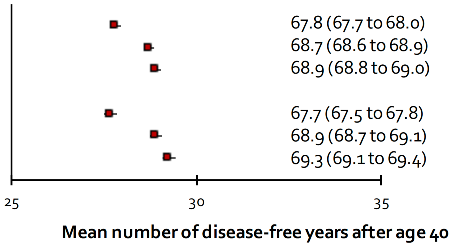

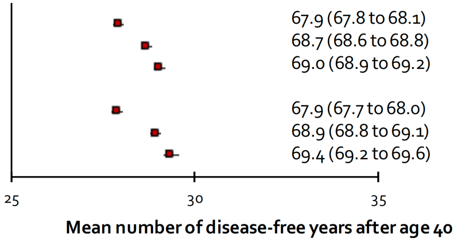

Figure S5. Association of leisure-time physical activity with disease-free years between ages 40 and 75 in men and women in the IPD-Work with and without restricting the age at baseline

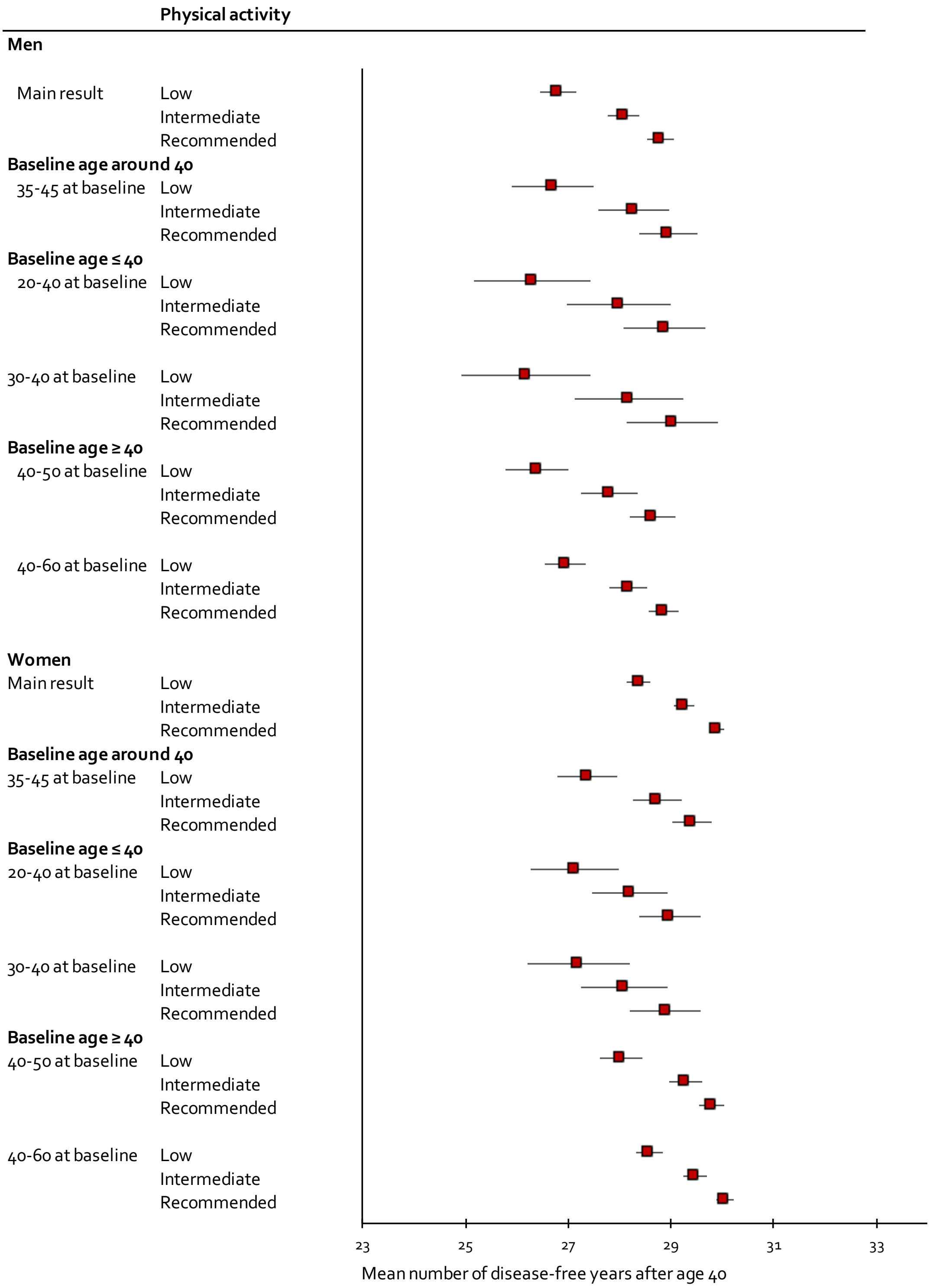

Figure S6. Association of leisure-time physical activity, MET and daily TV watching time with disease-free years between ages 40 and 75 in men and women in the IPD-Work and UK Biobank cohorts

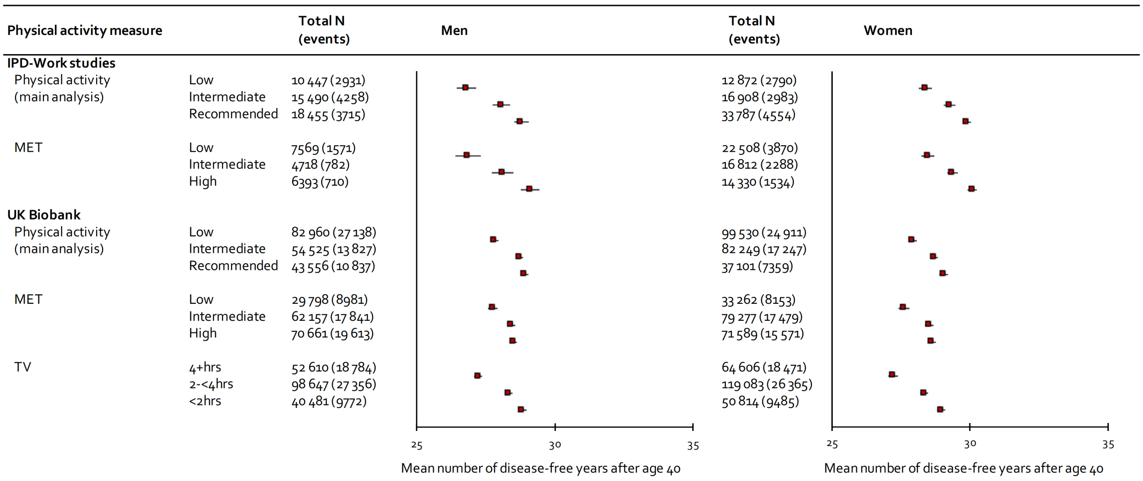

Figure S7. Association of leisure-time physical activity with disease-free years between ages 40 and 75 in men and women in the IPD-Work and UK Biobank cohorts by education and SES

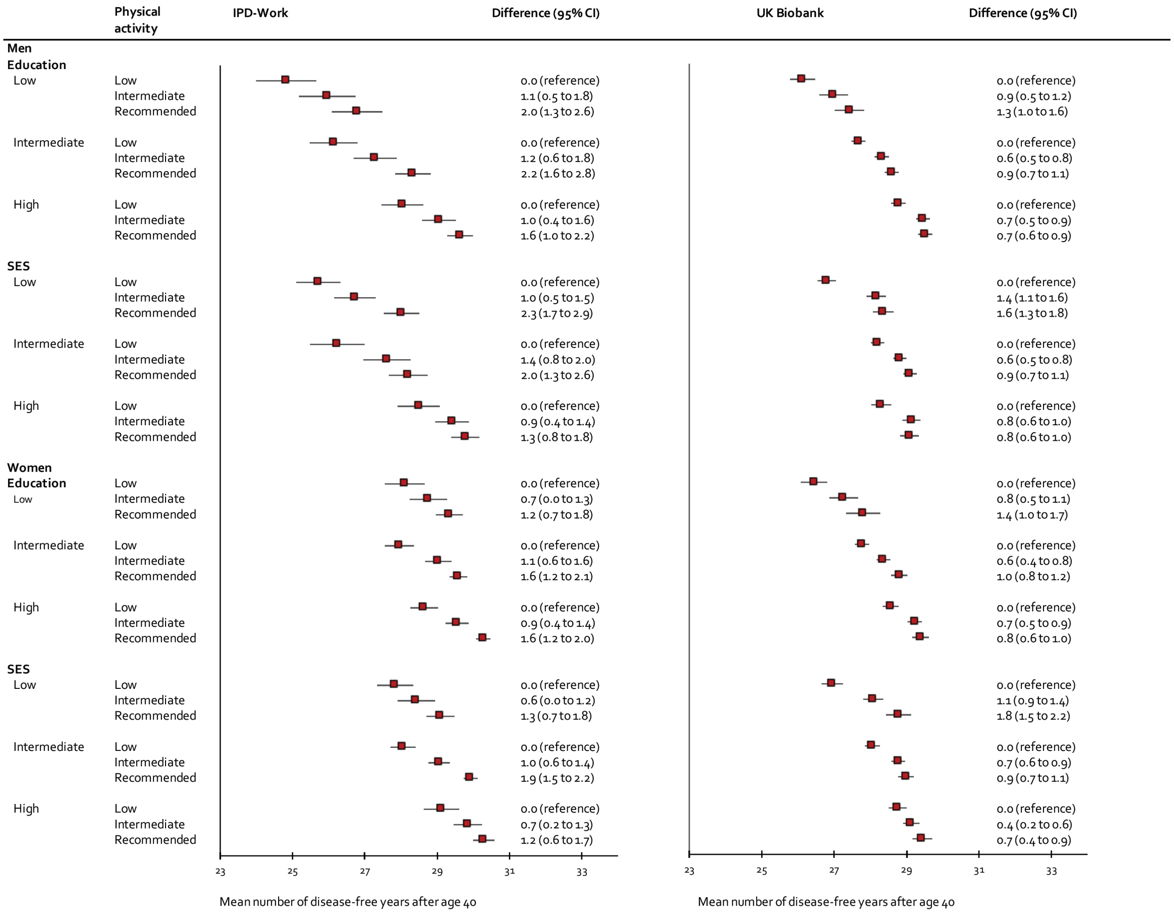

Figure S8. Association of leisure-time physical activity with disease-free years between ages 40 and 75 in men and women in the IPD-Work and UK Biobank cohorts by lifestyle categories

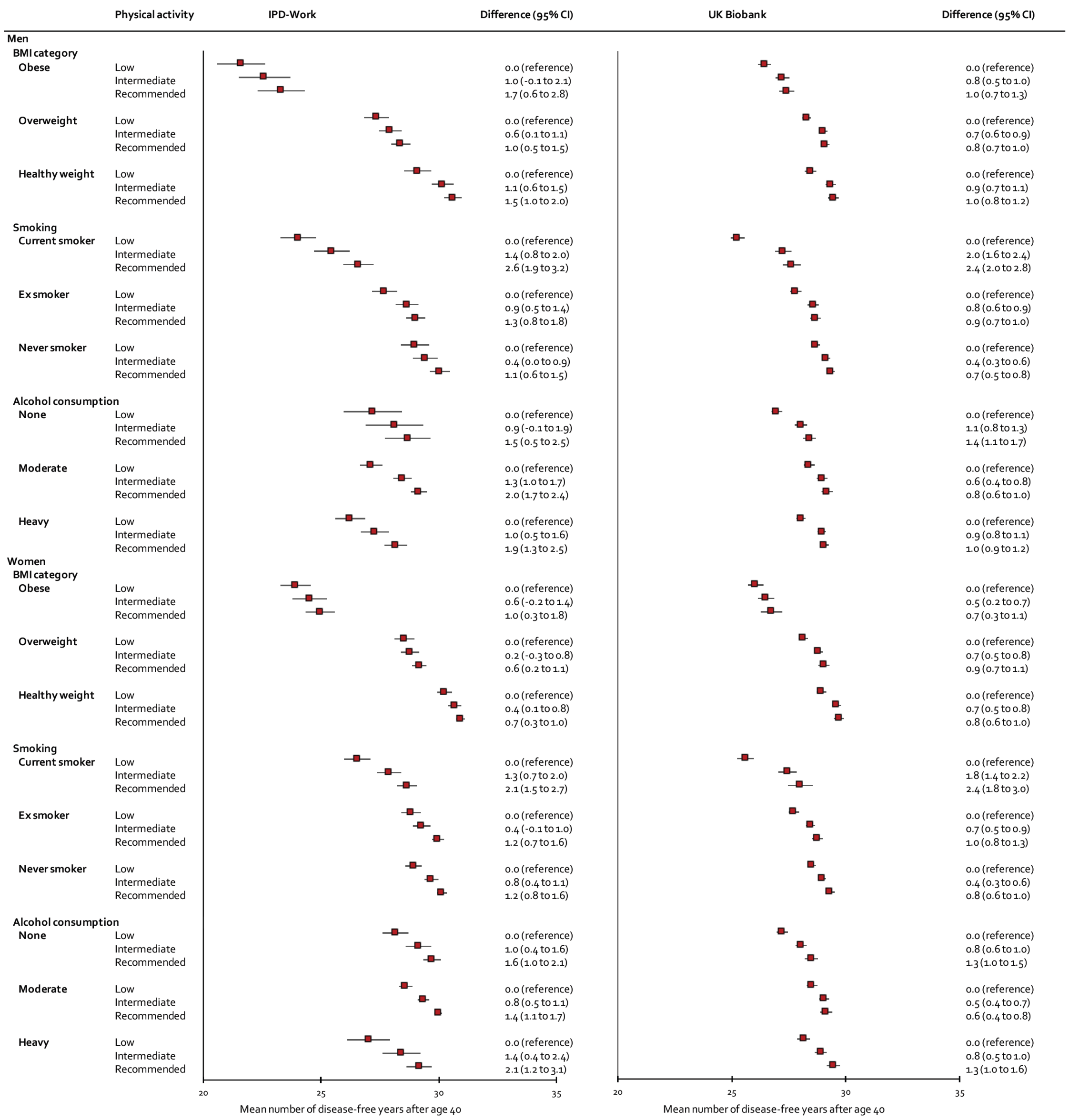

Figure S9. Association of leisure-time physical activity with disease-free years between ages 40 and 75 in men and women in the IPD-Work and UK Biobank cohorts by mental health status

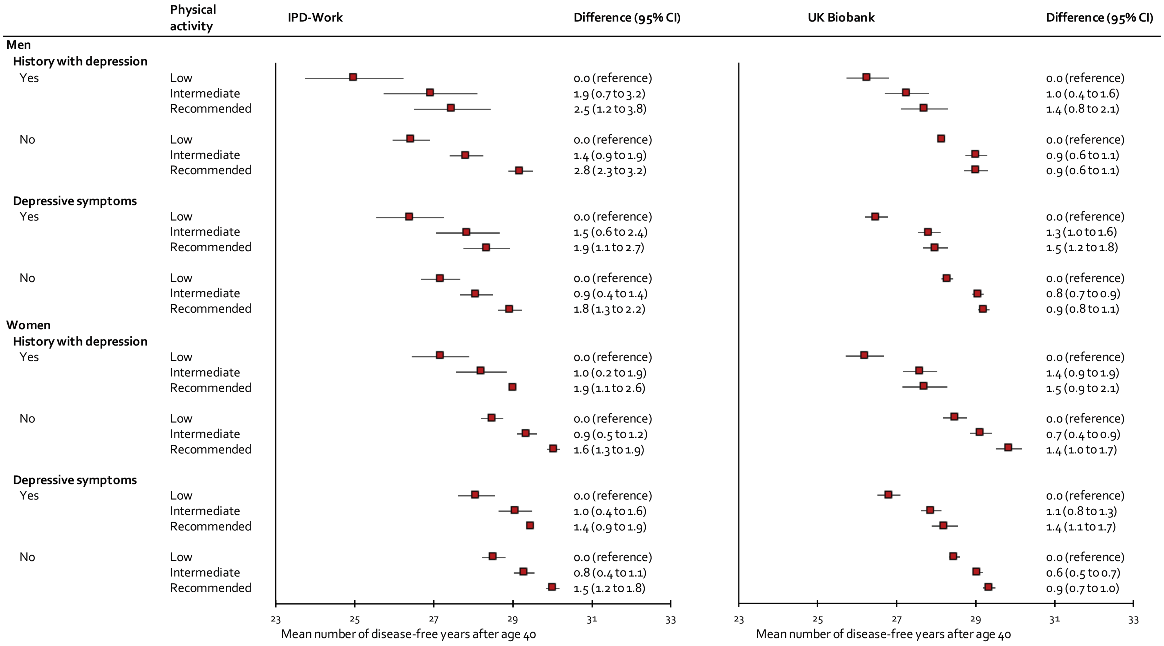

Figure S10. Association of leisure-time physical activity with disease-free years between ages 40 and 75 in men and women in the IPD-Work with different categorisation for alcohol consumption

	Physical activity	IPD-Work	Difference (95% CI)
**Men**			
**Alcohol consumption**			
**None**	Low		0.0 (reference)
	Intermediate		0.9 (-0.1 to 1.9)
	Recommended		1.5 (0.5 to 2.5)
**Moderate**	Low		0.0 (reference)
	Intermediate		1.3 (1.0 to 1.7)
	Recommended		2.0 (1.7 to 2.4)
**Heavy**	Low		0.0 (reference)
	Intermediate		1.0 (0.5 to 1.6)
	Recommended		1.9 (1.3 to 2.5)
**Alcohol consumption, alternative definition***			
**None**	Low		0.0 (reference)
	Intermediate		0.9 (-0.1 to 1.9)
	Recommended		1.5 (0.5 to 2.5)
**Moderate**	Low		0.0 (reference)
	Intermediate		1.2 (0.9 to 1.6)
	Recommended		2.0 (1.6 to 2.3)
**Intermediate**	Low		0.0 (reference)
	Intermediate		1.3 (0.1 to 2.5)
	Recommended		1.9 (0.7 to 3.2)
**Heavy**	Low		0.0 (reference)
	Intermediate		1.3 (0.4 to 2.2)
	Recommended		2.4 (1.4 to 3.4)
**Women**			
**Alcohol consumption**			
**None**	Low		0.0 (reference)
	Intermediate		1.0 (0.4 to 1.6)
	Recommended		1.6 (1.0 to 2.1)
**Moderate**	Low		0.0 (reference)
	Intermediate		0.8 (0.5 to 1.1)
	Recommended		1.4 (1.1 to 1.7)
**Heavy**	Low		0.0 (reference)
	Intermediate		1.4 (0.4 to 2.4)
	Recommended		2.1 (1.2 to 3.1)
**Alcohol consumption, alternative definition***			
**None**	Low		0.0 (reference)
	Intermediate		1.0 (0.4 to 1.6)
	Recommended		1.6 (1.0 to 2.1)
**Moderate**	Low		0.0 (reference)
	Intermediate		0.8 (0.5 to 1.1)
	Recommended		1.4 (1.1 to 1.7)
**Intermediate**	Low		0.0 (reference)
	Intermediate		0.6 (-1.0 to 2.3)
	Recommended		0.8 (-0.7 to 2.4)
**Heavy**	Low		0.0 (reference)
	Intermediate		1.7 (0.2 to 3.1)
	Recommended		2.5 (1.2 to 3.8)

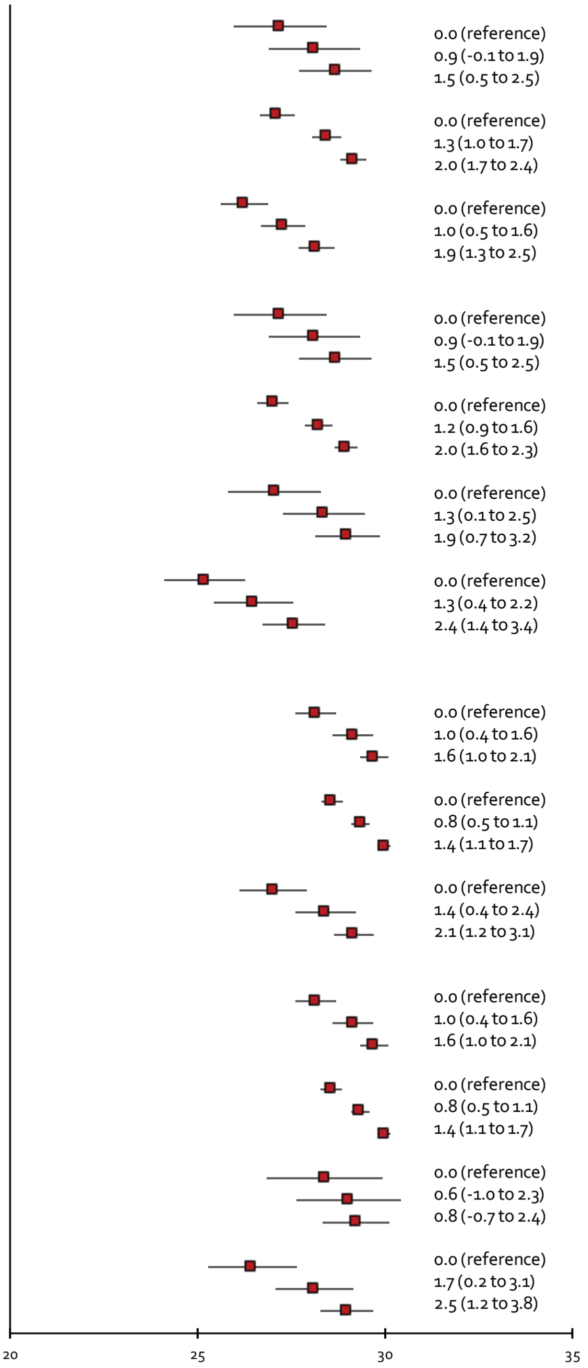

Figure S11. Comparison of disease-free years gained from recommended leisure-time physical activity between participants in the high-risk versus low-risk categories of socioeconomic, lifestyle factors and depression separately in the IPD-Work cohorts and UK Biobank

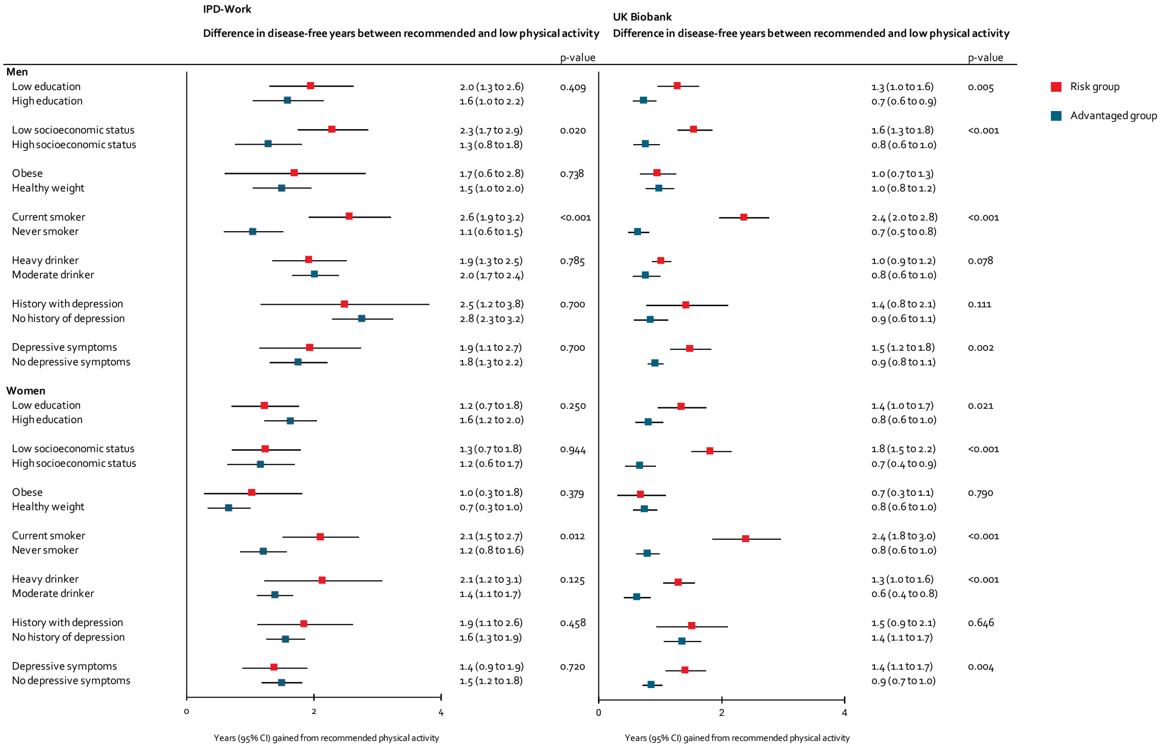

Extended statistical methods

Due to sex differences in the age of onset of chronic diseases, analyses were carried out separately for men and women. To evaluate the validity of self-reported physical activity, we compared accelerometer-measured mean levels of 24-hour physical activity between low, intermediate and recommended levels of self-reported physical activity

Due to small numbers in subgroups of individual cohorts and as in previous IPD-Work studies, we used pooled data in analysis of IPD-Work cohorts and added cohort study as a covariate in adjusted analyses.[2,24,58,59] We examined whether the use of pooled individual-level data across all studies was likely to be robust for studying subgroups. To do so, we compared results from the pooled analysis with those obtained from the two-stage meta-analysis. In the two-step meta-analysis approach, the first step was to determine cohort specific effects estimates and the latter step was to combine the estimates using fixed-effects and random-effects meta-analysis (table S3 below).

Table S3. Comparison of effect estimates between 1-step pooled analysis and 2-step individual-participant meta-analysis

	**Difference in disease-free years between recommended activity and low leisure-time physical activity (years)**		
**Population and level of physical activity**	**Pooled analysis**	**Fixed-effect meta-analysis**	**Random-effect meta-analysis**
Men			
Low	0.00 (reference)	0.00 (reference)	0.00 (reference)
Intermediate	1.3 (1.0 to 1.5)	1.1 (0.9-1.4)	1.1 (0.7-1.5)
Recommended	2.0 (1.7 to 2.3)	1.9 (1.6-2.2)	2.0 (1.3-2.7)
Women			
Low	0.00 (reference)	0.00 (reference)	0.00 (reference)
Intermediate	0.9 (0.6 to 1.2)	0.9 (0.6-1.2)	0.9 (0.6-1.2)
Recommended	1.5 (1.3 to 1.8)	1.6 (1.3-1.8)	1.5 (1.1-1.8)

We defined disease-free years as the number of life-years between ages 40 and 75 during which an individual was free from diagnosis of any of the six chronic diseases examined. We chose age 40 because this is typically the age at which health checks, monitoring of specific cancers, and cardiovascular risk assessments typically begin.

To estimate the associations between self-reported physical activity categories and disease-free years, hazard ratios (HR) with 95% confidence intervals (CI) for the first disease occurrence were calculated using flexible parametric survival models on the cumulative hazards scale.[60,61] Restricted cubic splines with 0 to 4 internal knots were fitted within these models to estimate the baseline hazard for each physical activity category using age as the timescale. The number of knots for each model was selected using the Akaike Information Criteria (AIC).[62] The number of knots in the main analysis and the subgroup models are shown in the table S4 below. As a sensitivity analysis we repeated the main analysis allowing for time-dependent effects for physical activity (Stata tvc command, table S5 below).

Table S4. The number of knots for models selected using the Akaike Information Criteria (AIC)

		IPD		UK Biobank	
		**Men**	**Women**	**Men**	**Women**
	**Main model:**	3	1	4	4
**Subgroup analyses**:	Category				
Education	Low	1	1	0	1
	Intermediate	2	4	3	2
	High	2	0	3	2
SES	Low	3	1	1	3
	Intermediate	3	0	4	3
	High	1	0	3	1
BMI	Obesity	1	4	3	3
	Overweight	2	0	4	1
	Healthy weight	3	4	2	4
Smoking	Current	2	1	0	1
	Ex	3	1	2	1
	Never	3	0	3	3
Alcohol	None	0	0	0	3
	Moderate	2	1	2	2
	Heavy	2	0	4	3
Depression history	Yes	2	0	0	2
	No	2	0	2	1
Depressive symptoms	Yes	2	0	2	3
	No	2	4	4	1

Table S5. Comparison of the effect estimates between the main analysis and a sensitivity analysis allowing for time-dependent effects for physical activity

	Mean number of disease-free years after age 40 (years)	
**Population and level of physical activity**	**Main analysis**	**Sensitivity analysis**
IPD-Work		
Men		
Low	26.8 (26.5 to 27.2)	26.6 (26.2 to 27.0)
Intermediate	28.1 (27.8 to 28.4)	27.8 (26.7 to 28.8)
Recommended	28.8 (28.5 to 29.1)	28.9 (28.3 to 29.5)
Women		
Low	28.4 (28.1 to 28.6)	28.3 (28.1 to 28.6)
Intermediate	29.3 (29.0 to 29.5)	29.1 (28.4 to 29.8)
Recommended	29.9 (29.8 to 30.0)	29.9 (29.5 to 30.2)
UK Biobank		
Men		
Low	27.8 (27.7 to 28.0)	27.6 (27.4 to 27.8)
Intermediate	28.7 (28.6 to 28.9)	28.9 (28.6 to 29.2)
Recommended	28.9 (28.8 to 29.0)	29.1 (28.4 to 29.8)
Women		
Low	27.9 (27.8 to 28.1)	27.7 (27.5 to 27.9)
Intermediate	28.7 (28.6 to 28.8)	29.1 (27.6 to 30.6)
Recommended	29.0 (28.9 to 29.2)	29.0 (27.7 to 30.3)

Age was used as the time scale in the analysis. The computation was based on the area under the curve within a pre-specified age range (40-75), with each participant contributing to the estimation from baseline at the time the participant responded to the questionnaire survey, irrespective of their age, to disease onset, death or end of follow-up, whichever occurred first. Consequently, the full age range was utilized to estimate the survival curves. At baseline, 70% of IPD Work participants and nearly all UK Biobank participants were at age 40 or older at baseline (see the table S6 below).

Table S6. The age distribution of participants at the baseline

Age	FPS	Gazel	HeSSup	HHS	Still working	WOLF N	WOLF S	Whitehall II	Total
**Men**									
**<40**	2529	0	4369	0	3241	1339	1379	33	12890
(%)	29.9	0	49.0	0	47.9	36.0	44.7	0.6	
**40+**	5920	6620	4542	1320	3523	2375	1706	5496	31502
(%)	70.1	100	51.0	100	52.1	64.0	55.3	99.4	
**Total**	8449	6620	8911	1320	6764	3714	3085	5529	44392
**Women**									
**<40**	10826	0	6885	0	791	240	989	14	19745
(%)	30.2	0	53.4	0	40.0	33.9	43.7	0.6	
**40+**	25036	2489	6016	4887	1188	468	1276	2462	43822
(%)	69.8	100	46.6	100	60.0	66.1	56.3	99.4	
**Total**	35862	2489	12901	4887	1979	708	2265	2476	63567

Disease-free years achieved in relation to physical activity categories compared with the reference group (low physical activity) were calculated as the difference between the areas under the disease-free survival curves from age 40 to age 75 years, (an age range used in previous studies[2,24,28]). Area under the curve was computed via numerical integration with a spline-based method. Disease-free years were estimated conditional on survival to age 40 without any of the six chronic diseases investigated. We repeated this analysis after including also kidney disease, liver disease and dementia in the list of chronic conditions. We chose age 40 because this is typically the age at which health checks, monitoring of specific cancers, and cardiovascular risk assessments begin.[63-65] Confidence intervals and p-values for disease-free years were estimated via bootstrapping using 1000 independent replications.

Robustness of the association between physical activity and disease-free life-years in the total study population was confirmed in sensitivity analyses repeating the main analysis while using different measures for physical activity, such as weekly MET hours and, as a proxy measure of sedentary behaviour, TV-watching time.

Subgroup analyses stratified by socioeconomic variables were conducted for education (low, intermediate or high) and adulthood socioeconomic status (low, intermediate or high). We also stratified analyses by the following lifestyle factors: BMI (healthy weight, overweight, obesity), smoking (current, ex or never), alcohol consumption (none, moderate or heavy drinking). Subgroup analyses by mental health status were based on two variables: history of depression (yes or no) and current depressive symptoms (yes or no). High-risk categories of these subgroup variables included low education, low SES, obesity, current smoking, heavy drinking, history of depression, and current depressive symptoms. In a sensitivity analysis alcohol intake was categorised as none, moderate (women: 1-14, men: 1-21 drinks/week), intermediate (women: 15-20, men: 22-27 drinks/week) and heavy (women: >20, men: >27 drinks/week)[29] The proportion of participants with missing data for education, SES, BMI, smoking, alcohol consumption or mental health status was relatively small. Therefore, those with missing data were excluded from the corresponding subgroup analyses.

To test the hypothesis that individuals with health risks or disadvantaged backgrounds may benefit more from physical activity than those with favourable conditions, we combined effect estimates from IPD and UK Biobank using random-effects meta-analysis and tested the statistical significance of the difference in physical activity-related gain in disease-free years between high-risk and low-risk groups using I2 statistics. We also used a conventional z-test for two independent samples to examine this difference; the p-values obtained from the I2 statistics and z-test were identical (see table S7 below).

Table S7. Comparison of disease-free years gained from recommended leisure-time physical activity between participants in the high-risk versus low-risk categories of socioeconomic, lifestyle factors and depression in the IPD-Work and UK Biobank datasets

Men	Difference in disease-free years between recommended activity and low activity (years)	p-value* I^2-test	p-value* z-test
Low education	1.434 (1.134 to 1.734)	0.001	0.001
High education	0.836 (0.656 to 1.016)		
Low socioeconomic status	1.712 (1.461 to 1.963)	<0.001	<0.001
High socioeconomic status	0.865 (0.666 to 1.064)		
Obese	1.014 (0.729 to 1.299)	0.753	0.753
Healthy weight	1.064 (0.940 to 1.188)		
Current smoker	2.423 (2.078 to 2.768)	<0.001	<0.001
Never smoker	0.697 (0.538 to 0.856)		
Heavy drinker	1.086 (0.934 to 1.238)	0.835	0.835
Moderate drinker	1.112 (0.920 to 1.304)		
History with depression	1.651 (1.056 to 2.246)	0.334	0.334
No history of depression	1.335 (1.095 to 1.575)		
Depressive symptoms	1.563 (1.256 to 1.870)	0.001	0.001
No depressive symptoms	0.990 (0.865 to 1.115)		
**Women**			
Low education	1.314 (0.998 to 1.630)	0.111	0.111
High education	1.010 (0.811 to 1.209)		
Low socioeconomic status	1.680 (1.400 to 1.960)	<0.001	<0.001
High socioeconomic status	0.770 (0.540 to 1.000)		
Obese	0.769 (0.419 to 1.119)	0.859	0.859
Healthy weight	0.735 (0.603 to 0.867)		
Current smoker	2.269 (1.860 to 2.678)	<0.001	<0.001
Never smoker	0.887 (0.718 to 1.056)		
Heavy drinker	1.365 (1.118 to 1.612)	0.003	0.003
Moderate drinker	0.910 (0.738 to 1.082)		
History with depression	1.650 (1.191 to 2.109)	0.464	0.464
No history of depression	1.461 (1.247 to 1.675)		
Depressive symptoms	1.409 (1.133 to 1.685)	0.010	0.010
No depressive symptoms	1.001 (0.859 to 1.143)		

The prevalence of the six chronic diseases increases with age. Since participants had to be free of these diseases at baseline, health-related selection may affect results from the UK Biobank study, in which participants were markedly older than those in the IPD-Work cohort studies. To investigate this possibility, we repeated the main analysis in UK Biobank cohort by age group.

Analyses were conducted using SAS 9·4 statistical software for Windows and Stata/MP 18·0 for Mac, package stpm2.[66]

Statistical code

An example of the code for calculating the estimate of the disease-free life-years in the pooled dataset

```
insheet using pooled_pasgp.txt, clear
gen expo=.
replace expo=0 if h_physact=="sedentary" // reference
replace expo=1 if h_physact=="intermediate"
replace expo=2 if h_physact=="active"

egen koho=group(study)

vl clear
vl set
vl substitute mjalist = (i.expo i.koho)


*define the survival data
stset loppupvm, failure(status_uh==1) id(id) origin(syntpvm) enter(basedate) exit(failure) scale(365.25)

*disease-free years are calculated between 40 and 75
egen t1 = seq(), f(4000) t(7500)
replace t1 = t1*0.01
summarize t1

*the model, knots are defined with df(). Df - 1 equals knots
capture noisily xi: stpm2 $mjalist, df(1) scale(hazard)

mat b=e(b)'

* Survival curves
* Predict survival curve and save data.
predict S0, survival zero time(t1)
* Normalize. The survival curve should start from 1
gen S0n = S0/S0[1]
* Estimate survival curves at each level of exposure
gen S1 = S0n^exp(0)
gen S2 = S0n^exp(b[1,1])
gen S3 = S0n^exp(b[2,1])
* Area under the curve. Integration is through cubic spline
integ S1 t1
gen iS1 = r(integral)
scalar LE_occ1 = iS1
integ S2 t1
gen iS2 = r(integral)
scalar LE_occ2 = iS2
integ S3 t1
gen iS3 = r(integral)
scalar LE_occ3 = iS3
```

drop S0 S1 S2 S3 iS* S0n

****
matrix YL = (LE_occ1, LE_occ2, LE_occ3)

*** *
Bootstrap to get the standard error and confidence interval
```
capture program drop myboot
program define myboot, rclass
 preserve
  bsample
  xi: stpm2  $mjalist , df(1) scale(hazard)
  mat b=e(b)'
  predict bS0, survival zero time(t1)
  sort t1 bS0
  gen bS0n = bS0/bS0[1]
  gen bS1 = bS0n^exp(0)
  gen bS2 = bS0n^exp(b[1,1])
  gen bS3 = bS0n^exp(b[2,1])

  integ bS1 t1
  gen ibS1 = r(integral)
  integ bS2 t1
  gen ibS2 = r(integral)
  integ bS3 t1
  gen ibS3 = r(integral)

  return scalar YL_1 = ibS1
  return scalar YL_2 = ibS2
  return scalar YL_3 = ibS3

  drop bS0 bS1 bS2 bS3 ibS1 ibS2 ibS3 bS0n

  *
  restore
end
```

*** Generate the bootstrap samples**
simulate YL_1=r(YL_1) YL_2=r(YL_2) YL_3=r(YL_3), reps(1000) seed(12345): myboot

*** Generate statistics table**
bstat, stat(YL)

*** Observed statistic**
mat theta = e(b)
*** Bootstrapped standard error**
mat theta_se = e(se)

****
gen LE_1 = theta[1,1]
gen LE_2 = theta[1,2]

```
gen LE_3 = theta[1,3]

gen seLE_1 = theta_se[1,1]
gen seLE_2 = theta_se[1,2]
gen seLE_3 = theta_se[1,3]
```

References

1. World Health Organization. WHO guidelines on physical activity and sedentary behaviour Geneva: World Health Organization; 2020. Licence: CC BY-NC-SA 3.0 IGO. 2020. Available at: https://www.who.int/publications/i/item/9789240015128 (accessed 30 January 2024.
2. Nyberg ST, Singh-Manoux A, Pentti J, et al. Association of Healthy Lifestyle With Years Lived Without Major Chronic Diseases. *JAMA Intern Med* 2020; **180**(5): 760-8.
3. Fransson EI, Heikkila K, Nyberg ST, et al. Job Strain as a Risk Factor for Leisure-Time Physical Inactivity: An Individual-Participant Meta-Analysis of Up to 170,000 Men and Women: The IPD-Work Consortium. *Am J Epidemiol* 2012; **176**(12): 1078-89.
4. Heikkila K, Fransson EI, Nyberg ST, et al. Job strain and health-related lifestyle: findings from an individual-participant meta-analysis of 118,000 working adults. *Am J Public Health* 2013; **103**(11): 2090-7.
5. Bonekamp NE, Visseren FLJ, Ruigrok Y, et al. Leisure-time and occupational physical activity and health outcomes in cardiovascular disease. *Heart* 2023; **109**(9): 686-94.
6. Holtermann A, Hansen JV, Burr H, Sogaard K, Sjogaard G. The health paradox of occupational and leisure-time physical activity. *Br J Sports Med* 2012; **46**(4): 291-5.
7. Holtermann A, Marott JL, Gyntelberg F, et al. Occupational and leisure time physical activity: risk of all-cause mortality and myocardial infarction in the Copenhagen City Heart Study. A prospective cohort study. *BMJ Open* 2012; **2**(1): e000556.
8. Karihtala T, Valtonen AM, Kautiainen H, et al. Relationship between occupational and leisure-time physical activity and the need for recovery after work. *Arch Public Health* 2023; **81**(1): 17.
9. Kivimäki M, Lawlor DA, Smith GD, et al. Socioeconomic Position, Co-Occurrence of Behavior-Related Risk Factors, and Coronary Heart Disease: the Finnish Public Sector Study. *Am J Public Health* 2007; **97**(5): 874-9.
10. Leskinen T, Stenholm S, Heinonen OJ, et al. Change in physical activity and accumulation of cardiometabolic risk factors. *Prev Med* 2018; **112**: 31-7.
11. Goldberg M, Leclerc A, Bonenfant S, et al. Cohort profile: the GAZEL Cohort Study. *Int J Epidemiol* 2007; **36**(1): 32-9.
12. Korkeila K, Suominen S, Ahvenainen J, et al. Non-response and related factors in a nation-wide health survey. *Eur J Epidemiol* 2001; **17**(11): 991-9.
13. Lahelma E, Aittomaki A, Laaksonen M, et al. Cohort profile: the Helsinki Health Study. *Int J Epidemiol* 2013; **42**(3): 722-30.
14. Väänänen A, Murray M, Koskinen A, Vahtera J, Kouvonen A, Kivimäki M. Engagement in cultural activities and cause-specific mortality: prospective cohort study. *Prev Med* 2009; **49**(2-3): 142-7.
15. Marmot MG, Smith GD, Stansfeld S, et al. Health inequalities among British civil servants: the Whitehall II study. *Lancet* 1991; **337**(8754): 1387-93.
16. Alfredsson L, Hammar N, Fransson E, et al. Job strain and major risk factors for coronary heart disease among employed males and females in a Swedish study on work, lipids and fibrinogen. *Scand J Work Environ Health* 2002; **28**(4): 238-48.
17. Peter R, Alfredsson L, Hammar N, Siegrist J, Theorell T, P. W. High effort, low reward, and cardiovascular risk factors in employed Swedish men and women: baseline results from the WOLF Study. *J Epidemiol Community Health* 1998; **52**: 540-7
18. Pulakka A, Leskinen T, Koster A, Pentti J, Vahtera J, Stenholm S. Daily physical activity patterns among aging workers: the Finnish Retirement and Aging Study (FIREA). *Occup Environ Med* 2019; **76**(1): 33-9.

19. van Hees VT, Gorzelniak L, Dean León EC, et al. Separating movement and gravity components in an acceleration signal and implications for the assessment of human daily physical activity. *PLoS One* 2013; **8**(4): e61691.
20. Stenholm S, Suorsa K, Leskinen T, et al. Finnish Retirement and Aging Study: a prospective cohort study. *BMJ Open* 2023; **13**(12): e076976.
21. Menai M, van Hees VT, Elbaz A, Kivimaki M, Singh-Manoux A, Sabia S. Accelerometer assessed moderate-to-vigorous physical activity and successful ageing: results from the Whitehall II study. *Sci Rep* 2017; **8**: 45772.
22. Kivimaki M, Nyberg ST, Batty GD, et al. Job strain as a risk factor for coronary heart disease: a collaborative meta-analysis of individual participant data. *Lancet* 2012; **380**(9852): 1491-7.
23. Kivimaki M, Strandberg T, Pentti J, et al. Body-mass index and risk of obesity-related complex multimorbidity: an observational multicohort study. *Lancet Diabetes Endocrinol* 2022; **10**(4): 253-63.
24. Nyberg ST, Batty GD, Pentti J, et al. Obesity and loss of disease-free years owing to major non-communicable diseases: a multicohort study. *Lancet Public Health* 2018; **3**(10): e490-e7.
25. Nyberg ST, Heikkilä K, Fransson EI, et al. Job strain in relation to body mass index: pooled analysis of 160 000 adults from 13 cohort studies. *J Intern Med* 2012; **272**(1): 65-73.
26. Heikkilä K, Nyberg ST, Fransson EI, et al. Job Strain and Tobacco Smoking: An Individual-Participant Data Meta-Analysis of 166 130 Adults in 15 European Studies. *PLoS One* 2012; **7**(7): e35463.
27. UK Chief Medical Officers. UK Chief Medical Officers' low risk drinking guidelines 2016. https://www.gov.uk/government/publications/alcohol-consumption-advice-on-low-risk-drinking (accessed Feb 12 2020.
28. Nyberg ST, Batty GD, Pentti J, et al. Association of alcohol use with years lived without major chronic diseases: A multicohort study from the IPD-Work consortium and UK Biobank. *Lancet Reg Health Eur* 2022; **19**: 100417.
29. Heikkilä K, Nyberg ST, Fransson EI, et al. Job Strain and Alcohol Intake: A Collaborative Meta-Analysis of Individual-Participant Data from 140 000 Men and Women. *PLoS One* 2012; **7**(7): e40101.
30. Goldberg DP, Gater R, Sartorius N, et al. The validity of two versions of the GHQ in the WHO study of mental illness in general health care. *Psychol Med* 1997; **27**(1): 191-7.
31. Virtanen M, Lallukka T, Ervasti J, et al. The joint contribution of cardiovascular disease and socioeconomic status to disability retirement: A register linkage study. *Int J Cardiol* 2017; **230**: 222-7.
32. Holi MM, Marttunen M, Aalberg V. Comparison of the GHQ-36, the GHQ-12 and the SCL-90 as psychiatric screening instruments in the Finnish population. *Nord J Psychiatry* 2003; **57**(3): 233-8.
33. Stansfeld SA, Marmot MG. Social class and minor psychiatric disorder in British Civil Servants: a validated screening survey using the General Health Questionnaire. *Psychol Med* 1992; **22**(3): 739-49.
34. Beck AT, Ward CH, Mendelson M, Mock J, Erbaugh J. An inventory for measuring depression. *Arch Gen Psychiatry* 1961; **4**: 561-71.
35. Nabi H, Kivimaki M, Suominen S, Koskenvuo M, Singh-Manoux A, Vahtera J. Does depression predict coronary heart disease and cerebrovascular disease equally well? The Health and Social Support Prospective Cohort Study. *Int J Epidemiol* 2010; **39**(4): 1016-24.
36. GBD 2017 Causes of Death Collaborators. Global, regional, and national age-sex-specific mortality for 282 causes of death in 195 countries and territories, 1980-2017: a systematic analysis for the Global Burden of Disease Study 2017. *Lancet* 2018; **392**(10159): 1736-88.

37. Disease GBD, Injury I, Prevalence C. Global, regional, and national incidence, prevalence, and years lived with disability for 354 diseases and injuries for 195 countries and territories, 1990-2017: a systematic analysis for the Global Burden of Disease Study 2017. *Lancet* 2018; **392**(10159): 1789-858.
38. Kivimaki M, Hamer M, Batty GD, et al. Antidepressant medication use, weight gain, and risk of type 2 diabetes: a population-based study. *Diabetes Care* 2010; **33**(12): 2611-6.
39. Tabak AG, Jokela M, Akbaraly TN, Brunner EJ, Kivimaki M, Witte DR. Trajectories of glycaemia, insulin sensitivity, and insulin secretion before diagnosis of type 2 diabetes: an analysis from the Whitehall II study. *Lancet* 2009; **373**(9682): 2215-21.
40. Alberti KG, Zimmet PZ. Definition, diagnosis and classification of diabetes mellitus and its complications. Part 1: diagnosis and classification of diabetes mellitus provisional report of a WHO consultation. *Diabet Med* 1998; **15**(7): 539-53.
41. Fransson EI, Nyberg ST, Heikkila K, et al. Job strain and the risk of stroke: an individual-participant data meta-analysis. *Stroke* 2015; **46**(2): 557-9.
42. Heikkila K, Nyberg ST, Theorell T, et al. Work stress and risk of cancer: meta-analysis of 5700 incident cancer events in 116,000 European men and women. *BMJ* 2013; **346**: f165.
43. Heikkila K, Madsen IE, Nyberg ST, et al. Job strain and COPD exacerbations: an individual-participant meta-analysis. *Eur Respir J* 2014; **44**(1): 247-51.
44. Heikkila K, Madsen IE, Nyberg ST, et al. Job strain and the risk of severe asthma exacerbations: a meta-analysis of individual-participant data from 100 000 European men and women. *Allergy* 2014; **69**(6): 775-83.
45. Nyberg ST, Fransson EI, Heikkila K, et al. Job strain as a risk factor for type 2 diabetes: a pooled analysis of 124,808 men and women. *Diabetes Care* 2014; **37**(8): 2268-75.
46. . https://www.ukbiobank.ac.uk/ (accessed February 3, 2022.
47. Guidelines for Data Processing and Analysis of the International Physical Activity Questionnaire (IPAQ)
– Short and Long Forms. https://biobank.ndph.ox.ac.uk/showcase/ukb/docs/ipaq_analysis.pdf (accessed 30 May 2023.
48. Townsend P, Phillimore P, Beattie A. Health and Deprivation: Inequality and the North: Croom Helm; 1988.
49. Smith DJ, Nicholl BI, Cullen B, et al. Prevalence and characteristics of probable major depression and bipolar disorder within UK biobank: cross-sectional study of 172,751 participants. *PLoS One* 2013; **8**(11): e75362.
50. UK Biobank Resource 158772: Derivation of mental states. https://biobank.ndph.ox.ac.uk/showcase/refer.cgi?id=158772 (accessed 18 Dec 2023.
51. UK Biobank Data-Field 2050: Frequency of depressed mood in last 2 weeks. https://biobank.ndph.ox.ac.uk/showcase/field.cgi?id=2050 (accessed 18 December 2023.
52. Doherty A, Jackson D, Hammerla N, et al. Large Scale Population Assessment of Physical Activity Using Wrist Worn Accelerometers: The UK Biobank Study. *PLoS One* 2017; **12**(2): e0169649.
53. Guo C, Tam T, Bo Y, Chang LY, Lao XQ, Thomas GN. Habitual physical activity, renal function and chronic kidney disease: a cohort study of nearly 200 000 adults. *Br J Sports Med* 2020; **54**(20): 1225-30.
54. Schneider CV, Zandvakili I, Thaiss CA, Schneider KM. Physical activity is associated with reduced risk of liver disease in the prospective UK Biobank cohort. *JHEP Rep* 2021; **3**(3): 100263.
55. Livingston G, Huntley J, Liu KY, et al. Dementia prevention, intervention, and care: 2024 report of the Lancet standing Commission. *Lancet* 2024; **404**(10452): 572-628.
56. Kivimaki M, Frank P, Pentti J, et al. Obesity and risk of diseases associated with hallmarks of cellular ageing: a multicohort study. *Lancet Healthy Longev* 2024; **5**(7): e454-e63.

57. Fraser HC, Kuan V, Johnen R, et al. Biological mechanisms of aging predict age-related disease co-occurrence in patients. *Aging Cell* 2022; **21**(4): e13524.
58. Nyberg ST, Batty GD, Pentti J, et al. Association of alcohol use with years lived without major chronic diseases: a multicohort study from the IPD-Work consortium and UK Biobank. *Lancet Reg Health Eur* 2022.
59. Kivimaki M, Kuosma E, Ferrie JE, et al. Overweight, obesity, and risk of cardiometabolic multimorbidity: pooled analysis of individual-level data for 120 813 adults from 16 cohort studies from the USA and Europe. *Lancet Public Health* 2017; **2**(6): e277-e85.
60. Royston P, Parmar MK. Flexible parametric proportional-hazards and proportional-odds models for censored survival data, with application to prognostic modelling and estimation of treatment effects. *Stat Med* 2002; **21**(15): 2175-97.
61. Andersson TM-L, Dickman PW, Eloranta S, Lambe M, Lambert PC. Estimating the loss in expectation of life due to cancer using flexible parametric survival models. *Stat Med* 2013; **32**(30): 5286-300.
62. Bozdogan H. Model selection and Akaike's Information Criterion (AIC): The general theory and its analytical extensions. *Psychometrika* 1987; **52**(3): 345-70.
63. Goff DC, Jr., Lloyd-Jones DM, Bennett G, et al. 2013 ACC/AHA guideline on the assessment of cardiovascular risk: a report of the American College of Cardiology/American Heart Association Task Force on Practice Guidelines. *J Am Coll Cardiol* 2014; **63**(25 Pt B): 2935-59.
64. Piepoli MF, Hoes AW, Agewall S, et al. 2016 European Guidelines on cardiovascular disease prevention in clinical practice: The Sixth Joint Task Force of the European Society of Cardiology and Other Societies on Cardiovascular Disease Prevention in Clinical Practice (constituted by representatives of 10 societies and by invited experts)Developed with the special contribution of the European Association for Cardiovascular Prevention & Rehabilitation (EACPR). *Eur Heart J* 2016; **37**(29): 2315-81.
65. Hellquist BN, Duffy SW, Abdsaleh S, et al. Effectiveness of population-based service screening with mammography for women ages 40 to 49 years: evaluation of the Swedish Mammography Screening in Young Women (SCRY) cohort. *Cancer* 2011; **117**(4): 714-22.
66. Lambert PC, Royston P. Further development of flexible parametric models for survival analysis. *Stata Journal* 2009; **9**(2): 265-90.

STROBE Statement

	Item No	Recommendation
**Title and abstract**	1	(*a*) Indicate the study's design with a commonly used term in the title or the abstract **pages 1,5**
		(*b*) Provide in the abstract an informative and balanced summary of what was done and what was found **pages 5–6**
**Introduction**		
Background/rationale	2	Explain the scientific background and rationale for the investigation being reported **pages 7–8**
Objectives	3	State specific objectives, including any prespecified hypotheses **page 8**
**Methods**		
Study design	4	Present key elements of study design early in the paper **page 8, Supplement, pages 2-4,7**
Setting	5	Describe the setting, locations, and relevant dates, including periods of recruitment, exposure, follow-up, and data collection **pages 8–9, Supplement pages 2-4,7, Table 1**
Participants	6	(*a*) Give the eligibility criteria, and the sources and methods of selection of participants. Describe methods of follow-up **Figure 1, Table 1, pages 8–9, Supplement pages 2-4,7**
		(*b*) For matched studies, give matching criteria and number of exposed and unexposed **N/A**
Variables	7	Clearly define all outcomes, exposures, predictors, potential confounders, and effect modifiers. Give diagnostic criteria, if applicable **pages 9–11, Supplement 5–8, 12–25**
Data sources/ measurement	8*	For each variable of interest, give sources of data and details of methods of assessment (measurement). Describe comparability of assessment methods if there is more than one group **pages 8–11, Supplement 5–8**
Bias	9	Describe any efforts to address potential sources of bias **pages 12-14**
Study size	10	Explain how the study size was arrived at **Figure 1, page 14-15**
Quantitative variables	11	Explain how quantitative variables were handled in the analyses. If applicable, describe which groupings were chosen and why **pages 9–11, Supplement 4–6**
Statistical methods	12	(*a*) Describe all statistical methods, including those used to control for confounding **pages 12-14, Supplement pages 35–40**
		(*b*) Describe any methods used to examine subgroups and interactions **pages 13-14, Supplement pages 35–38**
		(*c*) Explain how missing data were addressed **page 8, 14, Supplement page 5, 7, 38, Figure 1**
		(*d*) If applicable, explain how loss to follow-up was addressed **Supplement 6–8**
		(*e*) Describe any sensitivity analyses **pages 10-11, 16-17, Supplement pages 35–38**
**Results**		
Participants	13*	(a) Report numbers of individuals at each stage of study—eg numbers potentially eligible, examined for eligibility, confirmed eligible, included in the study, completing follow-up, and analysed **Figure 1, page 14-15, Supplement pages 2–4, 7**
		(b) Give reasons for non-participation at each stage **Figure 1, page 14-15, Supplement pages 2–4, 7**
		(c) Consider use of a flow diagram **Figure 1**
Descriptive data	14*	(a) Give characteristics of study participants (eg demographic, clinical, social) and information on exposures and potential confounders **Table 1, pages 14-15, Supplement 5–8, 12–25**
		(b) Indicate number of participants with missing data for each variable of interest **Figure 1, Supplement 12–25**
		(c) Summarise follow-up time (eg, average and total amount) **Table 1, page 15**
Outcome data	15*	Report numbers of outcome events or summary measures over time **Page 14-15, Figure 2**

Main results	16	(*a*) Give unadjusted estimates and, if applicable, confounder-adjusted estimates and their precision (eg, 95% confidence interval). Make clear which confounders were adjusted for and why they were included **Figure 2**
		(*b*) Report category boundaries when continuous variables were categorized **pages 9–11**
		(*c*) If relevant, consider translating estimates of relative risk into absolute risk for a meaningful time period **N/A**
Other analyses	17	Report other analyses done—eg analyses of subgroups and interactions, and sensitivity analyses **pages 15–17**
**Discussion**		
Key results	18	Summarise key results with reference to study objectives **page 17**
Limitations	19	Discuss limitations of the study, taking into account sources of potential bias or imprecision. Discuss both direction and magnitude of any potential bias **pages 20-21**
Interpretation	20	Give a cautious overall interpretation of results considering objectives, limitations, multiplicity of analyses, results from similar studies, and other relevant evidence **pages 17–21**
Generalisability	21	Discuss the generalisability (external validity) of the study results **pages 21**
**Other information**		
Funding	22	Give the source of funding and the role of the funders for the present study and, if applicable, for the original study on which the present article is based **pages 6, 14, 23**

*Give information separately for exposed and unexposed groups.

Note: An Explanation and Elaboration article discusses each checklist item and gives methodological background and published examples of transparent reporting. The STROBE checklist is best used in conjunction with this article (freely available on the Web sites of PLoS Medicine at http://www.plosmedicine.org/, Annals of Internal Medicine at http://www.annals.org/, and Epidemiology at http://www.epidem.com/). Information on the STROBE Initiative is available at http://www.strobe-statement.org.
